# Cellular and Molecular Mechanisms of Hypertrophy of Ligamentum Flavum

**DOI:** 10.3390/biom14101277

**Published:** 2024-10-10

**Authors:** Prashanta Silwal, Allison M. Nguyen-Thai, Peter G. Alexander, Gwendolyn A. Sowa, Nam V. Vo, Joon Y. Lee

**Affiliations:** 1 Ferguson Laboratory for Spine Research, Department of Orthopaedic Surgery, University of Pittsburgh, Pittsburgh, PA 15219, USA; 2 Department of Chemistry and Biochemistry, University of California, Los Angeles, CA 90095, USA; 3 Department of Physical Medicine and Rehabilitation, University of Pittsburgh Medical Cancer, University of Pittsburgh, Pittsburgh, PA 15261, USA

**Keywords:** lumbar spinal stenosis, ligamentum flavum, aging, TGF-β, low back pain, hypertrophy, fibrosis

## Abstract

Hypertrophy of the ligamentum flavum (HLF) is a common contributor to lumbar spinal stenosis (LSS). Fibrosis is a core pathological factor of HLF resulting in degenerative LSS and associated low back pain. Although progress has been made in HLF research, the specific molecular mechanisms that promote HLF remain to be defined. The molecular factors involved in the onset of HLF include increases in inflammatory cytokines such as transforming growth factor (TGF)-β, matrix metalloproteinases, and pro-fibrotic growth factors. In this review, we discuss the current understanding of the mechanisms involved in HLF with a particular emphasis on aging and mechanical stress. We also discuss in detail how several pathomechanisms such as fibrosis, proliferation and apoptosis, macrophage infiltration, and autophagy, in addition to several molecular pathways involving TGF-β1, mitogen-activated protein kinase (MAPKs), and nuclear factor-κB (NF-κB) signaling, PI3K/AKT signaling, Wnt signaling, micro-RNAs, extracellular matrix proteins, reactive oxygen species (ROS), etc. are involved in fibrosis leading to HLF. We also present a summary of the current advancements in preclinical animal models for HLF research. In addition, we update the current and potential therapeutic targets/agents against HLF. An improved understanding of the molecular processes behind HLF and a novel animal model are key to developing effective LSS prevention and treatment strategies.

## 1. Introduction

Lumbar spinal stenosis (LSS) is a common spine condition in aging populations and is one of the most common reasons for surgical intervention. LSS can be congenital, acquired or both with acquired ones being the major observed form of LSS [1]. Hypertrophy of the ligamentum flavum (HLF) is one of the main contributors in addition to disc degeneration, facet degeneration, ligamentum flavum (LF) calcification, spondylolisthesis, etc. causing acquired LSS [1,2]. The LF is an important anatomical structure covering most of the posterolateral part of the lumbar spinal canal. It is thickest at the L4–L5 spine level [3,4,5], with the tendency to thicken with increasing age [3,4], and the thickening is associated with disc degeneration and disc herniation [4]. Histologically, normal LF is composed predominantly of elastic fibers; however, HLF shows an increased proportion of collagen fibers and reduced elastic fibers [6], leading to fibrosis, a common cause of HLF [7,8,9,10].

**Figure 1:**
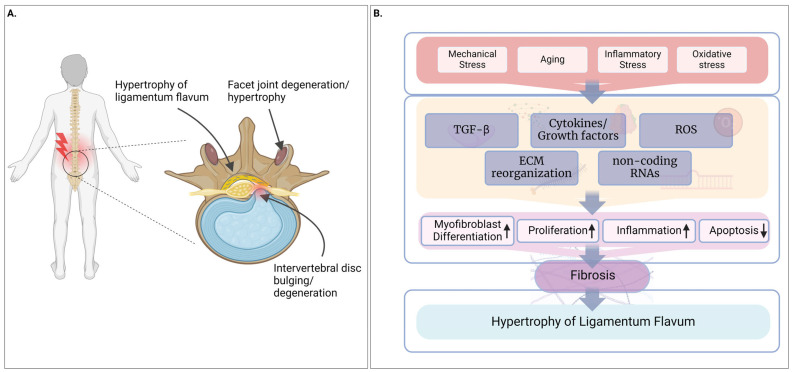
General outlook of lumbar spinal stenosis (LSS) and hypertrophy of ligamentum flavum (HLF). (**A**) HLF along with hypertrophy of facet joint and intervertebral disc bulging, or degeneration contributes to narrowing of the spinal canal causing lumbar spinal stenosis (LSS). (**B**) The reported stress, signaling molecules, and consequences of fibrosis and HLF that cause LSS are shown. Mechanical stress in addition to aging and associated inflammatory or oxidative stress initiates the fibrotic changes mediated through myofibroblast differentiation, inflammation, proliferation, and inhibition of apoptosis regulated through molecular signaling such as transforming growth factor (TGF)-β, other cytokines and growth factors, reactive oxygen species (ROS), non-coding RNAs, etc. These signaling events cause the accumulation of extracellular matrix (ECM) and fibrosis leading to development of HLF. Please refer to text for detailed explanations. (Figure created with BioRender.com, accessed on 1 August 2024).

Fibrosis is a core pathological factor of HLF resulting in degenerative LSS and associated low back pain [7]. Although the exact pathomechanism of HLF has not been identified, it is found that mechanical stress and aging are closely associated with hypertrophy [10]. The factors involved in the onset of HLF include increases in the expression of inflammatory cytokines, matrix metalloproteinases (MMPs), and pro-fibrotic growth factors [11,12]. HLF is mediated through signaling molecules/pathways regulating fibrosis and proliferation [13,14,15,16]. HLF is closely related to pro-fibrotic marker transforming growth factor-beta (TGF-β) [15]. The induction of TGF-β signaling is mediated through the SMAD pathway which leads to the increased expression of collagen I (COLI), collagen III (COLIII), TGF-β1, versican (VCAN), as well as upregulation of cell differentiation into myofibroblasts [17,18,19,20,21]. A brief overview of LSS and HLF is presented in Figure 1.

As explained, HLF is associated with the fibrosis of LF often leading to an increase in its thickness; however, its pathogenesis is still not clear. Also, LSS caused by the thickening of LF due to wild-type transthyretin amyloid deposition rather than fibrotic changes are also reported [22]. In most of the reported studies, ‘LF thickening’ and ‘hypertrophy of LF’ are used interchangeably, so one should be careful not to confuse them as being the same [4]. In this review, we are explaining HLF as the major cause of LSS and are mentioning LF thickening if the reported studies have mentioned it in their reports. Further research is required to provide a comprehensive understanding of this issue.

Here, we discuss recent research updates and advances in our understanding of the pathological and molecular mechanisms behind HLF, focusing on LF fibrosis and the associated mechanisms.

## 2. Overview of LSS and HLF

Lower back pain (LBP) is the leading cause of years-lost-to-disability, costing an estimated 0.1–2% of GDP in industrialized countries [23,24]. One of the leading causes of lower-back-related disability is LSS. It is a primary cause of neurogenic claudication, or the inability to walk normally due to the compression of the cauda equina (lower back nerve roots). LSS affects an estimated 30 million persons in the United States alone [25,26,27]. LSS is also the most common reason for spinal surgery in patients over the age of 65 [27,28,29]. 

HLF is the leading anatomic abnormality found in LSS. Surgical treatments are geared toward removing the HLF via various modalities. The laminectomy procedure is currently the gold standard surgical treatment for LSS. Despite many recent advancements in surgical treatment, there is still considerable room for improvement [27,28]. In 2007, laminectomy, the most frequent operative intervention for LSS, was attributable to $1.65 billion in Medicare costs [30]. Current evidence shows that non-operative measures have mixed efficacies [31,32]. 

There are no FDA-approved preventative or restorative therapies for HLF [33]. This, in part, is due to a lack of coordinated understanding of the biological transformation that allows HLF to occur, as well as lack of an effective reliable animal model to study LSS. Therefore, a comprehensive understanding of the pathobiology of HLF and the development of a novel model of LSS is vital to gain fundamental scientific insights for the effective treatment associated with LSS.

## 3. Risk Factors Associated with the Development of HLF

### 3.1. Aging 

LSS is one of the common spinal disorders in patients over the age of 50 [10,34,35]. Studies have identified a high correlation between LF thickness and increasing age [10,36]. Analysis in patients with an age ranging from 10 to 85 years showed an increase in thickness with age at all the levels, with the most pronounced increase at L3–L4 and L4–L5 [10]. Another study also found that the thickness of the LF was increased with age where the thickness of the LF was measured at the L3–4, L4–5, and L5–S1 levels on both sides using the magnetic resonance imaging (MRI) of 200 patients in three different age groups: 21–40 years, 41–60 years, and 61–80 years [3]. Similarly, a study of 63 patients showed that the LF thickness (medial and lateral) increased by age group of 40s and 50s [37]. Overall, most of the studies identified aging as a major risk factor in the increased LF thickness and the development of LSS; however, the association between aging and HLF is poorly understood.

### 3.2. Disc Degeneration

LF thickness can also be significantly correlated with low back pain symptoms [37]. The study showed that low back pain subjects had thicker medial and lateral thickness, and the medial and lateral thickness of the LF had low to moderate and a moderate and significant correlation with the disc height and disc degeneration grade, respectively [37]. Although the sample size in the study was small, it provided vital information that the LF thickening increases disc degeneration. This may indicate that HLF is a reactive phenomenon due to an abnormal mechanical load, as discussed further below [37]. Another study showed that the mean thickness of the LF in patients with grade IV to V disc degeneration was higher than in those with grade I to III [4] and correlated with age and disc herniation. This suggested that the thickening of the LF may be due to its buckling into the spinal canal secondary to disc degeneration more than hypertrophic transformation [4]. However, Sakamaki et al. concluded, by using 162 LBP patients’ data, that LF thickening at the L4–L5 level starts at the age of 30–39, and that the thickening of the LF was not buckling of the LF into the spinal canal with disc degeneration as there was no correlation between the LF thickness and decrease in disc height [36]. In addition, a study involving 39 patients with a normal L4–L5 disc height showed that the mean thickness of the LF at L4–L5 was increased with a higher grade of disc degeneration [6]. The thickness was positively correlated with the age, segmental angulation of the spine, and grade of disc degeneration, suggesting that aging-related segmental instability leads to HLF [6]. This study is particularly important because they excluded the LF buckling by taking the LF thickness data from patients with a normal disc height to purely investigate the HLF. Similarly, Abbas, Janan et al. showed by using 65 LSS and 150 non-LSS patients that the LF thickness is age-dependent, revealing that L3–L4 and L4–L5 lumbar segments are more susceptible to increased LF thickness than L5–S1 [5]. However, a study by Safak et al. showed that the LF thickness is not correlated with aging in an analysis of 320 patients (age range of 21 to 82 years) [38]. Overall, studies identified that an age-dependent and/or disc degeneration-independent or -dependent increase in the LF thickness is a major cause of LSS; however, the buckling of the LF due to disc degeneration rather than an increased thickness of the LF as the cause of LSS is also reported [4,5]. 

### 3.3. Mechanical Stress

Mechanical stress along with aging is one of the major risk factors for HLF. As fibrosis is a type of scarring due to injury, scar formation in HLF might be due to a stress-related injury [9]. Mechanical stress leads to microinjury, which then induces chronic inflammation and tissue fibrosis [10]. A biomechanical study using a 3D finite element model showed that mechanical stress especially at the dorsal side of the LF causes tissue damage and the associated repairing process within the LF causing scar formation and tissue fibrosis [10]. In addition, mechanical stress induces TGF-β1 to promote collagen synthesis [39]. Several in vitro experiments have revealed that mechanical stress leads to the activation of fibrotic signaling in LF cells [40,41]. Cyclic stretch caused apoptosis in LF cells via the induction of reactive oxygen species (ROS) and caspase-9 [42]. Using a multi-torsional mechanical stretch stress loading device, Kwon et al. showed that the mechanical stress to LF cells enhances the production of inflammatory cytokines and angiogenic factors, and extracellular matrix (ECM)-regulating enzymes [43]. In addition, several animal models also revealed the involvement of mechanical stress in the development of HLF. A rabbit model showed that long-term mechanical stress causes elastic fiber disruption and matrix production leading to HLF [44]. The mechanical stress was produced by L2–3 and L4–5 posterolateral fusion with instrumentation and resection of the L3–4 supraspinal muscle, producing mechanical stress concentration with segmental instability at the L3–4 level [44]. Comparable results were observed in a mouse model of LF instability [45], revealing mechanical stress as one of the major contributing factors in the pathogenesis of HLF. Details on the animal models are explained in Section 8. 

## 4. Pathomechanisms Associated with HLF

### 4.1. ECM Remodeling and Fibrosis

Fibrosis is one of the major pathomechanisms involved in HLF which is marked by the loss of elastic fibers and increase in collagen [8,10]. The ECM plays a key role in the repair of injuries in several kinds of tissues including muscles, and the correct remodeling of the ECM is essential for the healing process [46]. An excessive accumulation of ECM components leading to an aberrant wound-healing response defines fibrosis [47]. During fibrosis and scar formation, ECM synthesis is often mediated by myofibroblasts [48]. Fibrosis is often associated with inflammatory responses, where immune cells including neutrophils are recruited at the site of injury releasing chemoattractant cytokines causing further infiltration of other immune cells including monocytes and macrophages [49]. The activation of macrophages leads to increased TGF-β1 which activates resident fibroblasts in the injured tissue to induce their differentiation and the inhibition of apoptosis leading to excessive ECM deposition and fibrosis [50,51].

**Figure 2:**
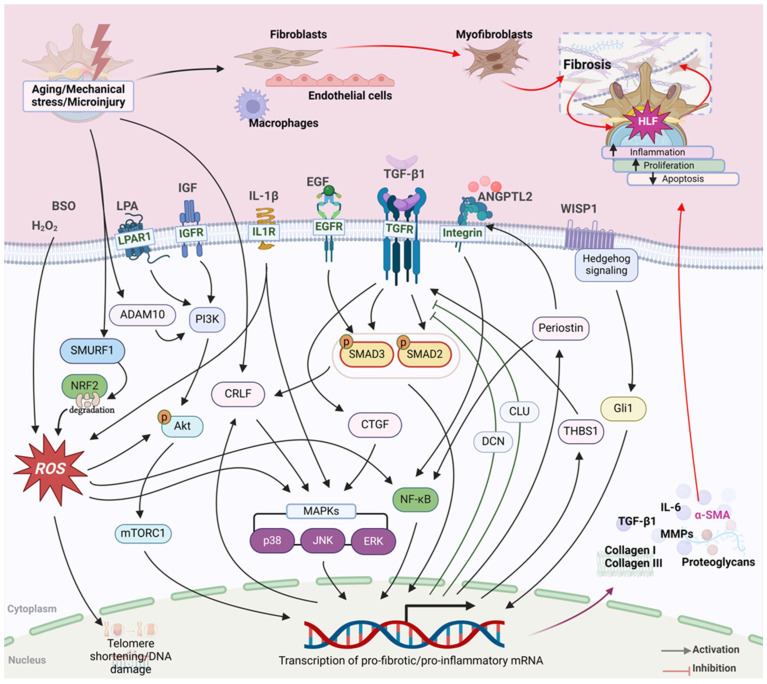
Proposed signaling pathways involved in the pathogenesis of hypertrophy of ligamentum flavum (HLF) based on reported findings. Mechanical stress/aging/microinjuries are the major factors inducing HLF through several signaling pathways leading to increase in inflammation, proliferation and ECM deposition and reduction in apoptosis thereby causing HLF. Transforming growth factor (TGF)-β1 signaling through TGF-β receptor (TGFR) is one of the main signaling events involved. Mechanical stress activates the inflammatory reaction at site of injury attracting macrophages, which along with endothelial cells and fibroblast releases the TGF-β1. TGF-β1 triggers the downstream signaling via TGFR to activate SMAD2 and SMAD3, which are then responsible for transcription of pro-inflammatory, pro-fibrotic markers such as interleukin (IL)-6, TGF-β1, matrix metallopeptidases (MMPs), Collagen I (COLI), COLIII, alpha-smooth muscle actin (α-SMA), etc. These factors further amplify the fibrotic process through cytokine receptor-mediated signaling to induce fibrosis and aid the pathogenesis of HLF. Specifically, α-SMA is involved in the myofibroblast differentiation of fibroblasts to increase the ECM components and fibrosis. Epidermal growth factor (EGF) also mediates the SMAD3-mediated signaling through the EGF receptor (EGFR). Increased level of connective tissue growth factor (CTGF) and periostin through TGF-β1 signaling induces fibrotic events by mitogen-activated protein kinases (MAPKs) and nuclear factor-κB (NF-κB) activation, respectively. In addition, periostin functions via the integrin receptor. Increased level of angiopoietin line 2 (ANGPTL2) during the HLF also activates Integrin α4β5-mediated NF-κB signaling. Furthermore, cytokine receptor-like factor 1 (CRLF) induced by TGF-β1 or IL-1β activates ERK MAPK to induce the transcription of pro-fibrotic genes. Accumulation of reactive oxygen species (ROS) occurs due to oxidative stress, inflammatory stress, or mechanical stress. An increase in ROS activates MAPK or NF-kB or phosphatidylinositol 3-kinase (PI3K)/AKT/mammalian target of the rapamycin (mTOR) signaling to induce fibrotic changes. Degradation of antioxidant protein nuclear factor erythroid 2-related factor 2 (NRF2) via SMURF1 increases the oxidative stress, further amplifying the fibrotic process. Also, increased ROS is associated with telomere shortening and DNA damage in HLF. In addition, PI3K/AKT/mTOR signaling is also activated by insulin-like growth factor-1 (IGF) (through IGFR), lysophosphatidic acid (LPA) (through LPAR), and A disintegrin and metalloproteinase 10 (ADAM10). Activation of Hedgehog-Gli1 signaling by Wnt1-inducible-signaling pathway protein 1 (WISP1) induces the expression of pro-fibrotic genes. Thrombospondin-1 (THBS1) activates TGF-β1-induced pro-fibrotic signaling. Further, decorin (DCN) and clusterin (CLN), which are induced by mechanical stress, are involved in negative regulation of TGF-β1-induced fibrotic signaling. For clarity, only some selected signaling pathways are included in this figure. Please refer to text for details. (Figure created with BioRender.com, accessed on 1 August 2024).

In the LF, scar formation and tissue fibrosis are identified as a pathology of HLF [10]. The increased expression of TGF-β from endothelial cells is thought to be the reason behind fibrosis in LF tissue leading to its hypertrophy [10]. The analysis of human LF samples showed severe fibrosis (scar formation) in HLF, and the severity of scarring was shown to be positively correlated with the LF thickness [9]. Further analysis revealed a weak but positive correlation between cyclooxygenase-2 (COX-2) expression in vascular endothelial cells in the LF to its thickness [9], suggesting an association between inflammatory responses to fibrosis in HLF. Myofibroblasts are increased in HLF tissues as compared to non-hypertrophic LF, suggesting the pivotal role of myofibroblast in the pathogenesis of HLF [17,52]. The expression of TGF-β1 and alpha smooth muscle actin (α-SMA), a marker for myofibroblast differentiation, has a positive correlation with LF thickness [52]. Exogenous TGF-β1 upregulated α-SMA and COLI, suggesting the trans-differentiation of fibroblasts into myofibroblasts [52]. A more recent study showed that myofibroblasts are increased in the dorsal layer of hypertrophic LF as identified by α-SMA positive cells [17]. This study suggested hypoxia as a cause of the increased myofibroblasts in the LF as cells cultured under hypoxic conditions increased the α-SMA gene expression [17]. These observations suggest that the transition of fibroblasts into myofibroblasts could be a major signaling event during the pathological change observed during the fibrosis of the LF. Based on the reported findings, we summarize the possible pathomechanisms and molecular pathways in Figure 2. 

### 4.2. Proliferation and Apoptosis 

Cell proliferation and cell apoptosis play a significant role in the wound-healing responses and fibrosis responses [53,54]. Increased cell proliferation and reduced apoptosis is thought to be one of the factors involved in the pathogenesis of HLF [55,56]. A study showed that the proliferation of LF cells is more common in HLF [57]. LF cell proliferation is found to be controlled by several signaling pathways/molecules such as lysophosphatidic acid (LPA) [57], A disintegrin and metalloproteinase 10 (ADAM10) [13], Hedgehog-Gli1 signaling [56], Acyl-CoA synthetase medium chain family member 5 (ACSM5) [55], insulin-like growth factor-1 (IGF-1) [58], and macrophage migration inhibitory factor (MIF) [59]. All the studies have identified that the above-mentioned signaling events promote cell proliferation and inhibit apoptotic signaling in LF cells thereby enhancing collagen deposition and LF fibrosis. Although the exact molecular interaction between cell proliferation, apoptosis, and LF fibrosis is not clear yet, the available results show that increased fibroblast proliferation and reduced apoptosis play significant roles in the pathogenesis of HLF.

### 4.3. Macrophage Infiltration

Macrophages are one of the major cell types other than fibroblasts that contribute to the pathophysiology of fibrotic diseases in several organs such as the lungs, kidneys, liver, skin, and heart [60,61]. Macrophage-secreted cytokines and growth factors have been implicated in the activation of fibroblasts to enhance fibrosis [62,63,64]. These secreted cytokines and growth factors such as TGF-β1 further enhance fibrosis by increasing the density of macrophages in injured tissue [51]. As in other fibrotic diseases, macrophage infiltration is reported in LF hypertrophy [65]. In the dorsal layer of the LF, an increased level of macrophage infiltration is observed as compared to the dural layer [65]. Using a microinjury-induced LF hypertrophy mouse model, Saito et al. showed that clodronate-lip-mediated macrophage depletion inhibits LF hypertrophy and collagen accumulation, suggesting that macrophage infiltration leads to fibroblast stimulation and fibrosis/HLF [65]. Further, macrophage migration inhibitory factor (MIF) is shown to promote the pathogenesis of HLF through Src kinase [59], suggesting the involvement of macrophage infiltration and migration in the development of HLF. Although M1-type macrophages are involved in the ossification of the LF [66,67], the exact origin and type of macrophage and/or fibroblast is not clear in the context of HLF. However, these studies showed the significant role of macrophages in the pathogenesis of HLF which in the future needs detailed attention.

### 4.4. Autophagy

Autophagy is a highly conserved self-renewal process involved in the clearance of aggregated proteins and damaged organelles through autophagosome–lysosome-mediated degradation. The role of autophagy in the pathogenesis of fibrotic diseases such as cardiac fibrosis, pulmonary fibrosis, and renal fibrosis is well studied [68,69,70]; however, there are few reports of the contribution of autophagy in HLF pathogenesis. Although several autophagy-related pathways such as PI3K/AKT/mTOR have been reported to be associated with HLF, the direct correlation of autophagy with LF pathogenesis is not studied in detail. A study showed that autophagy is activated in HLF tissues as revealed by the increased expression of beclin1 and LC3 and the reduced expression of p62 [71]. Mechanistically, VEGFA-mediated autophagy activation through miR-302b-3p is involved in HLF development [71]. Similar results were reported in a bioinformatic analysis which showed that several differentially expressed circular RNAs functioned in biological processes among which autophagy and mTOR signaling were top signaling pathways upregulated in HLF tissues as compared to non-hypertrophic LF tissues [72]. However, Li et al. [73], performed an HLF microarray analysis to reveal that autophagy-related biological pathways were downregulated whereas the ECM and collagen-related pathways were upregulated in HLF [73]. A validation study showed reduced autophagosomes and beclin1, and increased p62 expression in HLF tissues as compared to non-HLF tissues [73]. A study also showed comparable results from a mouse model of HLF, validating the bioinformatic results with human and mouse tissues [73]. This study is in discrepancy with the above-mentioned studies [71,72], suggesting a dual regulatory function of autophagy in HLF as is the case in renal fibrosis [74] and pulmonary fibrosis [68]. In the future, more studies must be conducted in different patient populations and animal models to clarify the role of autophagy in the pathogenesis of HLF. 

## 5. Molecular Signaling/Mechanisms Involved in HLF

### 5.1. TGF-β and Other Pro-Inflammatory Mechanisms

#### 5.1.1. TGF-β

The TGF-β family of cytokines acts as a key regulator of cell differentiation, migration, and proliferation, and is involved in tissue homeostasis and injury repairs in multiple cell types [51,75]. There are three closely related isoforms of the TGF-β family, and TGF-β1, -β2, and -β3 have important roles in the regulation of several cellular functions [75], with TGF-β1 being an important regulator of fibroblast phenotype and function [76]. The Golgi apparatus synthesizes each isoform from a disulfide-linked dimeric precursor [75]. The induction of signaling responses by TGF-β is mediated through TGF-β receptors (TGFBRs) resulting in ligand-induced transcription [77]. The receptors classified as type I and type II have a similar structural organization [77]. The best-known intracellular effectors in TGF-β signaling are SMAD proteins [78] acting through a canonical signaling pathway; and the presence of SMAD-independent signaling is reported [79]. 

As TGF-βs are known to mediate tissue fibrosis [75,80] its involvement in HLF is extensively reported (Figure 2). The level of TGF-β is higher in LF tissues from LSS patients as compared to non-LSS patients, revealing its involvement in HLF [16,81,82,83]. The concentration of TGF-β1 in the LF from LSS was found to be higher as compared to that from the LF from control patients with disc herniation [16,59]. Histological analysis from several studies has identified a higher expression of TGF-β in HLF [15,82,83]. A study showed that the expression of TGF-β is significantly higher in patients with HLF (an LF thickness of more than 4 mm) than the control (a thickness of the LF of less than 4 mm) [15]. Analysis also revealed that HLF patients have significantly more positively stained fibroblasts [15]. Other studies, however, showed that TGF-β1 was positively stained on the fibroblasts in the LF from both LSS and disc herniation patients [16], and there was no difference in TGF-β1 mRNA expression [52] between LSS and disc herniation patients, revealing a discrepancy in the reported role of TGF-β in the pathogenesis of HLF. In addition, the serum level of TGF-β and tissue inhibitor of matrix metalloproteinase (TIMP)-1 and TIMP-2 was not changed between LSS patients and the control (disc herniation), suggesting the local role of TGF-β1 rather than a systemic phenomenon [84]. A study to analyze the relationship between the relative expression of TGF-β mRNA and the thickness of the LF revealed a negative linear correlation, and only 8 of the 18 samples showed positive staining for TGF-β [10]. As the expression peaked in the mildly hypertrophied state and decreased in the well-hypertrophied state, the study suggested that TGF-β may contribute to hypertrophy only in the initial stages of the process [10]. Additionally, the expression of TGF-β1 is found to be positively correlated with COLI and COLIII and α-SMA expression in LF tissues, suggesting the critical role of TGF-β1 signaling in LF fibrosis leading to HLF [52]. In addition, there are some contrasting results regarding the source of TGF-β1 in HLF. Many of the studies have shown a higher expression of TGF-β1 in LF fibroblasts [15,16,59,83]. Löhr et al. reported that macrophages as well as vascular endothelial cells but not fibroblasts are the major sources for TGF-β1 [82]. Macrophages as well as fibroblasts are known sources of TGF-β1 in several fibrotic diseases [85,86], and the role of both the cell types in HLF cannot be denied; however, this needs detailed attention in the future to characterize the cell types that are responsible for TGF-β1 production during HLF pathogenesis. 

Studies have shown that the modulation of the TGF-β pathway regulates fibrotic signaling although the mechanism of activation and action are only partly revealed. LF tissues from LSS and non-LSS patients revealed that LF tissues from LSS patients express a higher level of TGF-β1 and phosphorylated SMAD3, suggesting that activated TGF-β signaling promotes the synthesis of COLI and COLIII through epidermal growth factor (EGF) during hypertrophy [81]. Exogenous EGF exposure enhanced the expression of phospho-EGFR, TGF-β1, and phospho-SMAD3; and TGF-β1-neutralizing antibody blocked the expression of phospho-SMAD3, COLI, and COLIII, suggesting that EGF signaling along with TGF-β1 signaling contributes to HLF [81]. The stimulation of LF cells with TGF-β1 induced the phosphorylation of SMAD2 but not SMAD3 and SMAD4, and knocking down SMAD2 led to the decreased expression of fibrotic genes, suggesting the role of SMAD2-mediated TGF-β signaling in HLF [87]. TGF-β1 in association with the increased expression of connective tissue growth factor (CTGF) led to p38 mitogen-activated protein kinase (MAPK) activation to induce HLF [88]. The suppression of TGF-β signaling by the CRISPR-mediated knockdown of TGFBR1 inhibited the myofibroblast differentiation in LF cells, revealing the importance of TGF-β-mediated signaling in the pathogenesis of HLF [89]. Overall, although partial knowledge exists, TGF-β1 and its associated signaling pathways have potential implications in the pathogenesis of HLF and can be a major therapeutic target. Based on these reported findings, summarized molecular pathways are presented in Figure 2.

#### 5.1.2. Interleukin-1β, Interleukin-6, and Tumor Necrosis Factor-α

Interleukin-1β (IL-1β), IL-6, and tumor necrosis factor-α (TNF-α) are potent proinflammatory cytokines involved in the host defense against infection and injury. They are known for their role in the innate and adaptive immune response and the regulation of fibrosis-related diseases and are potential therapeutic targets for several inflammatory disorders [90,91,92,93,94,95]. Although an increase in inflammatory cytokines such as IL-1β, IL-6, and TNF-α has been reported in intervertebral disc pathologies and aging-associated disorders [96,97,98], there are few studies to suggest the expression level of these cytokines in HLF. Sairo et al. reported that IL-1β and TNF-α are expressed in a comparable manner in hypertrophied and non-hypertrophied LF tissue [9]. However, recently, Ito et al. showed that the expression of TNF-α is significant but weakly correlated with the cross-sectional area of the LF [99]. These discrepancies between the studies highlight the need for more detailed studies to clearly identify the relationship between these pro-inflammatory cytokines and HLF. In experimental settings, however, inflammatory cytokines are used to induce the pro-fibrotic signaling in LF cells [100,101,102]. In LF cells, IL-1β and TNF-α stimulation caused the induction of COLI, COLIII, α-SMA, MMP2, and MMP-9 [101,102]. In addition, IL-1β promoted osteogenesis and inhibited adipogenesis in LF-derived cells, suggesting its role in stem cell lineage differentiation [103]. Similarly, the TNF-α level is increased in LF cells by mechanical stress or TGF-β [104], suggesting the role of TNF-α in LF fibrosis and the pathogenesis of HLF.

The expression of IL-6 mRNA is significantly higher in LF tissues from LSS patients than those from non-LSS patients [105]. The study showed that IL-6 mRNA expression was positively correlated with the LF thickness, suggesting its role in the pathogenesis of HLF and LSS [105]. A localization study showed the presence of IL-6 in the cytoplasm of vascular endothelial cells and the fibroblast-like cells of LF tissue [106]. Although the report did not show a direct correlation of IL-6 with the LF thickness, it suggested the IL-6/JAK/STAT signaling pathway activation in LF tissue, especially on the dorsal side rather than the dural side, revealing the activation of JAK/STAT signaling by IL-6 as a potential mechanism behind HLF [106]. The report also suggested the lumbar facet joint as a main source of IL-6 in LF tissues [106], which shows that multiple and complex crosstalk between several anatomical parts of the spine is involved in the pathogenesis of HLF. Also, Angiopoietin-like protein 2 (ANGPTL2) promotes inflammation in LF tissue by upregulating IL-6 expression and secretion (Figure 2) [105]. Mechanistically, ANGPTL2 mediated the nuclear translocation of NF-kB via integrin α5β1 to induce IL-6 expression in LF cells [105]. In addition, the expression of IL-6 was induced with mechanical stress, TGF-β, chemokines, and leptin in LF cells [107,108]. These studies suggest that the modulation of proinflammatory cytokines may provide a beneficial effect in relieving HLF and spinal stenosis, but their role in the pathogenesis of the LF is poorly understood. A summary of these reports is presented in Figure 2.

#### 5.1.3. Cytokine Receptor-like Factor 1 (CRLF1)

In addition to the cytokines such as TNF-α, IL-1β, and IL-6, a protein belonging to the cytokine receptor family has been implicated in the development of HLF. Cytokine receptor-like factor 1 (CRLF1), a secreted protein belonging to the cytokine type I receptor family, has a highly conserved WSXWS motif along with a hematopoietin domain with two fibronectin type III modules [109]. Multiomics analysis identified the increased expression of CRLF1 in HLF tissue as compared to normal LF, which was further confirmed by immunohistochemical analysis which showed that CRLF1 was expressed higher in HLF myofibroblasts [101]. Further analysis showed that the TGF-β1-induced SMAD3 pathway was involved in the regulation of CRLF1 expression and mediated the fibrosis through ERK signaling (Figure 2) [101]. Using an AAV vector system in vivo, it was found that CRLF1 suppresses the HLF formation in a bipedal standing mouse model. Although the exact source of CRLF1 expression is not known, the author discussed that mechanical stress and increased cytokines (IL-1β) and TGF-β1 could promote the transcription of CRLF1 thereby inducing pro-fibrotic signaling through ERK activation [101]. 

### 5.2. Reactive Oxidative Species

Normally, the generation of reactive oxygen species (ROS) through various processes is neutralized by enzymatic and non-enzymatic antioxidants; however, any imbalance in this process leads to the accumulation of ROS at the cellular level leading to oxidative stress [110]. During aging, there is an increase in ROS production along with decreased antioxidant defense leading to various aging-associated diseases [111]. Increased ROS further activates specific redox-sensitive signaling pathways and can induce strong inflammatory and pro-fibrotic signaling in multiple organs such as the heart, kidneys, lungs, and liver [112,113,114,115,116,117]. A study found that the levels of ROS radicals (superoxide anion (O_2_^•−^) and H_2_O_2_) along with malondialdehyde (MDA) and nitrotyrosine were higher in the LF from LSS patients, whereas the expression and level of antioxidant glutathione peroxidase (GPx-1), glutathione (GSH), and superoxide dismutase (SOD) activity were lower in the LF from LSS patients [14], suggesting a redox imbalance during HLF. Microarray analysis revealed that several genes associated with mitochondrial dysfunction are upregulated in HLF tissue [118]. As mitochondrial function is essential for the redox balance [119], a study showed that mitochondrial dysfunction is associated with an increase in oxidative stress as shown by the higher level of ROS and MDA content, and low level of the activity of the antioxidants, GSH and SOD, in the LF from LSS patients as compared to the LF from a disc herniation control group [118]. In LF cells, treatment with H_2_O_2_ increased the inflammatory, fibrotic, and apoptotic signaling through JNK and the p38 MAPK pathway as well as AKT signaling pathway [14]. Similarly, the tissue concentration of 8-hydroxy 2′-deoxygaunosine (8-OhdG) was found to be significantly higher in hypertrophic LF as compared to non-hypertrophic LF tissues, where the relative telomere length was lower in HLF tissue as compared to non-HLF tissues [120], suggesting that oxidative stress may lead to telomere shortening/DNA damage, contributing to the pathogenesis of HLF. In addition, another study found that the expression of 8-OhdG showed a weak but significant correlation with the cross-sectional area of the LF [99]. In addition, catalase, an antioxidant enzyme, is lower in LF tissues from LSS patients as compared to lumbar disc herniation patients [121]. Many antioxidants’ enzyme activity is controlled by nuclear factor-erythroid 2-related factor 2 (Nrf2) to protect the cells/tissues from oxidative stress-induced damage [122,123]. In HLF, a study found a reduced level of Nrf2 as compared to the LF tissues from disc herniation patients, and a mechanistic study found that an E3 ubiquitin-protein ligase, SMURF1, promoted the ubiquitination and degradation of Nrf2 leading to fibrosis and oxidative stress in LF cells [124], suggesting the role of redox imbalance in the pathogenesis of HLF. These studies suggest that mechanical stress or aging-associated changes cause a redox imbalance and increased oxidative stress in the LF and cause chronic inflammation and fibrosis, supporting the notion that like other aging-associated pathologies, in HLF too, a redox imbalance and increased oxidative stress could be one of the major etiological factors of LF hypertrophy. This makes mitochondrial function and oxidative stress potential therapeutic targets of LF hypertrophy and LSS symptoms. Based on these reported findings, a summarized molecular pathway is presented in Figure 2.

### 5.3. ECM Proteins

#### 5.3.1. Small Leucine-Rich Proteoglycans

The accumulation of the ECM and fibrosis is associated with the increased level of several glycoproteins. Biglycan (BGN), a family member of small leucine-rich proteoglycans (SLRPs), was expressed higher in the LF from a LSS group as compared to the control group [18]. As BGN is known to bind to collagen in the ECM [125], it is suggested that BGN can directly lead to HLF by increasing the ECM [18]. In addition, an in vitro study suggested that BGN can promote LF cell proliferation and myofibroblast differentiation [18]. Similarly, another member of SLRPs, decorin (DCN), was upregulated in HLF tissues [126]; however, external DCN treatment inhibited LF cell proliferation and TGF-β1 induced fibrosis in LF cells. DCN alleviated LF fibrosis in an animal model of HLF [126]. This study suggests the notion that increased DCN may participate in the repair process rather than the pathogenesis of HLF [126]. Another glycoprotein clusterin (CLU) also showed a similar pattern, as the CLU level was higher in HLF tissues and was induced by TGF-β1 in LF cells, but an external CLU inhibited the TGF-β1-induced fibrotic signaling in LF cells and prevented mechanical stress-induced HLF in vivo [41]. Mechanistically, CLU inhibits the phosphorylation of SMAD3 by competitively binding to the cell surface receptor, Activin receptor-like kinase 5 (ALK5), and the in vitro level of CLU was stabilized by protein kinase D3 (PRKD3) through the inhibition of lysosomal degradation [41]. 

#### 5.3.2. Thrombospondin-1

Thrombospondin-1 (THBS1), a calcium-sensitive protein secreted as a disulfide-linked homotrimer is essentially found in the ECM and is inducible by mechanical stress and fibrotic signaling. Differentially expressed protein analysis in LF tissues found that THBS1 was highly associated with fibrosis, with a protein–protein interaction network showing an interaction between TGFβ1 and THBS1 [127]. In vitro and in vivo studies found that mechanical stress-induced HLF is promoted through the THBS1/TGFβ1/SMAD3 signaling axis, which could be inhibited by Sestrin2, a stress-induced protein [127]. These studies suggest that several ECM proteins are induced during the development of HLF, with some of them serving to promote the repair and healing process. It is important in the future to understand the possible cross-link between different ECM proteins and their effect in the fibrosis process during the development of HLF. 

#### 5.3.3. CCN Family

The CCN family is composed of six matrix proteins (CCN1–6) which are matricellular proteins that modify the signaling associated with the extracellular matrix and play prominent roles in fibrotic diseases through the regulation of ECM remodeling and wound healing among other regulatory functions in inflammation, tumor development, etc. [128,129]. Connective tissue growth factor (CTGF, also known as CCN2), a cysteine-rich mitogen secreted by human vascular endothelial cells, is involved in normal physiological processes such as embryogenesis and differentiation and in several fibrotic disorders [130]. The CTGF expression was higher in LF tissues from LSS patients compared to the control group and the CTGF concentration exhibited a strong positive correlation with the LF thickness [88]. An in vitro study showed that recombinant CTGF can induce the expression of profibrotic markers such as COLI and COLIII in LF cells, suggesting its role in the pathogenesis of HLF [130]. 

Another CCN family protein, Wnt1-inducible-signaling pathway protein 1 (WISP1, also known as CCN4), is extensively involved in the fibrotic process [129]. In LSS patients, the expression of WISP-1 in LF tissue is higher, and recombinant WISP-1 in LF cells upregulated the expression of COLI and COLIII, suggesting a key role of WISP-1 in the pathogenesis of LF hypertrophy [56,131]. A study showed that WISP-1 expression is induced by mechanical stress in LF fibroblasts to promote LF cell proliferation and inhibit apoptosis [56]. Further results revealed that WISP-1 induces Hedgehog-Gli signaling to induce α-SMA thereby inducing mechanical stress-induced fibrosis and hypertrophy in vitro and in vivo [56], suggesting a critical role of the WISP-1/Hedgehog signaling axis in the development of HLF.

In contrast, CCN5 has been known to exert anti-fibrotic and anti-hypertrophic functions in cardiac tissues [132]. In the context of the HLF too, the stimulation of LF cells with CCN5 reduced the TGF-β1-induced pro-fibrotic markers such as α-SMA, COLI, COLIII, fibronectin, and CCN2 [89]. Although the study did not show any mechanistic studies or in vivo results, based on previous results on cardiac fibrosis [132], CCN5 could be evaluated as a therapeutic target against HLF. Based on these reported findings, the summarized pathway is included in Figure 2.

### 5.4. MAPK and NF-κB Signaling

MAPKs are serine/threonine kinases in multiple signal transduction systems that convert extracellular stimuli into a wide range of cellular responses and play important roles in a vast array of cellular processes such as inflammation, cell differentiation, division, and death [133]. MAPKs are activated upon several signaling cascades including TGF-β1 [134]. The best-known MAPKs include the extracellular signal-regulated kinases 1 and 2 (ERK1/2), c-Jun amino-terminal kinases (JNK), and p38 [135]. In LF cells, TGF-β1 [88] or TNF-α [100] stimulation induced the phosphorylation of p38 leading to pro-fibrotic and pro-inflammatory changes. Mechanistically, TGF-β1 stimulation stimulated the expression of CTGF leading to disturbed ECM homeostasis through the p38 MAPK pathway [88]. In addition, an increase in oxidative stress during aging and mechanical stress activated p38 and JNK MAPK signaling pathways to trigger inflammation and fibrosis of the LF leading to hypertrophy [14,136]. These reports highlighted that MAPKs might be key mediators of the effects of TGF-β1 and other inflammatory signaling to induce pro-fibrotic activities.

NF-κB, a family of inducible transcription factors, regulates a large array of genes involved in multiple physiological and pathological processes including fibrosis [137,138,139]. In HLF, although a direct correlation between hypertrophy and NF-κB is not yet understood, several inflammatory or oxidative stress signals are known to induce pro-fibrotic signaling via NF-κB activation [14,99]. In LF cells, oxidative stress signaling induced by H_2_O_2_ increases the phosphorylation of NF-κB through AKT or JNK and p38 MAPK signaling to induce inflammation and fibrosis [14]. Also, the treatment of LF cells with another oxidative stress inducer, BSO, induced fibrotic signaling through NF-κB and MAPK-mediated inflammatory reactions [99], highlighting the involvement of NF-κB-mediated signaling in HLF. In several disease models, crosstalk between TGF-β1 signaling and NF-κB is reported [140,141,142]; however, there are no such studies conducted in relation to HLF which requires attention in the future to determine the role of NF-κB in HLF. Based on these reports, the summarized pathway is presented in Figure 2.

### 5.5. PI3K/AKT/mTOR Signaling

PI3K/AKT/mTOR signaling is a fundamental signaling network in the cells involved in cell growth and proliferation, motility, metabolism, survival, etc. [143]. Differentially Expressed Gene (DEG) analysis has shown that the PI3K-AKT signaling pathway is among the most enriched biological pathways in ligamentum hypertrophy [144]. IGF-1 stimulation of LF cells led to the activation of mTORC1 signaling [58]. Also, a high AKT phosphorylation level was detected in LF tissues from LSS patients [58] and in LF cells under mechanical stress [145]. Zhou et al. showed that AKT activation through the lysophosphatidic acid receptor 1 (LPAR1)-mediated signaling pathway is involved in HLF [57]. LPAR1 expression in hypertrophic tissues and cells from HLF tissues was significantly higher than the non-hypertrophic group; and the treatment of LF cells with LPA induced proliferation, whereas apoptosis was inhibited through the regulation of the cyclin dependent kinase 1 (CDK1)/Cyclin B pathway. Mechanistically, LPA-LPARI stimulated AKT phosphorylation through mTORC2 to enhance cell survival [57]. Equivalent results were observed in a rat model of HLF, where Kil6425, an LPAR1 inhibitor, prevented LPA-induced LF hypertrophy through the inhibition of AKT phosphorylation [57]. Also, the expressions of LPA and LPAR1 were found to be associated with fibrosis and HLF in LSS patients [7]. Similarly, ADAM10 has been shown to increase the cell proliferation and hypertrophy in LF cells through the PI3K/AKT pathway [13]. Silencing and overexpression studies in human LF cells confirmed that ADAM10 promotes the proliferation of LF cells leading to LF hypertrophy via the PI3K/AKT pathway [13]. These studies identified that PI3K/AKT pathway activation is most probably associated with the mechanical stress contributing to HLF by inducing proliferation and inhibiting the apoptosis of LF cells. This makes PI3K/AKT a therapeutic target as there are several established inhibitors which are under clinical trials for several diseases including cancers, which can potentially be used in LF hypertrophy [146]. Figure 2 includes these reported findings.

### 5.6. Growth Factors

Growth factors are diffusible proteins that act within a short range from the site of production to activate diverse signaling pathways regulating a variety of cellular functions including cell proliferation [147]. Growth factor signaling mediated by several tyrosine kinase receptors and serine-threonine kinase receptors via fibroblast growth factor (FGF), EGF, vascular endothelial growth factor (VEGF), platelet-derived growth factor (PDGF), bone morphogenetic proteins (BMPs), and TGF-β promotes or suppresses cell proliferation and differentiations based on the tissue type and diseases such as lung or kidney fibrosis [147]. Increased levels of IGF-1 [58], EGF [81], and VEGF-1 [148] are associated with HLF. 

IGF-1 and IGF-1R expression along with AKT, S6, COLI, and COLIII were expressed significantly higher in the LF from LSS patients than that in non-LSS patients [58]. The treatment of LF cells with IGF-1 induced the phosphorylation of AKT and S6 which were blocked by the IGF-1R specific inhibitor, NVP-AEW541, and the mammalian target of the rapamycin C1 (mTORC1) specific inhibitor, rapamycin, suggesting the involvement of IGF-1 in the pathogenesis of LF hypertrophy via AKT/mTOR signaling [58]. Similarly, in vitro results showed that mechanical stress promotes IGF-1, COLI, and COLIII to induce the phosphorylation of IGF1R, AKT, and S6 in LF cells, which were blocked by IGF-1 neutralizing antibody or the inhibitor, NVP-AEW541 [145]. Although the study did not reveal the correlation between the expression of IGF-1/IGF-1R and the LF thickness, these results provide the important notion that mechanical stress-induced IGF-1 may promote LF cell proliferation leading to their hypertrophy. 

EGF, a single-chain polypeptide, triggers several biological responses including cell proliferation and differentiation, and is involved in several fibrotic diseases including airway, liver, and cardiac fibrosis [149,150,151]. In the LF, the expression of EGF and phospho-EGFR was higher in the LSS group as compared to non-LSS [81]. In LF cells, stimulation with EGF activates TGF-β1, COLI, and COLIII as well as the phosphorylation of SMAD3. Further, TGF-β1 neutralizing antibody and erlotinib, an EGFR-specific inhibitor, both blocked EGF-induced COLI and COLIII in LF cells suggesting the involvement of EGF in the regulation of fibrotic signaling in LF [81]. Although there is no established correlation between EGF and fibrotic signaling in the context of LF hypertrophy, based on studies in pathologies such as cancers and lung fibrosis [152,153], understanding the EGF signaling in the LF could foster the development of novel therapeutic strategies in the future. 

The mRNA and protein expression of VEGF, a key mediator of angiogenesis, is higher in the LF from LSS patients as compared to that from non-LSS LF [148]. Although a correlation between the thickness of the LF and the expression of VEGF is not reported, this study provides potential evidence that the VEGF may contribute to the pathogenesis of HLF. Transcriptomic analysis showed that FGF along with the Rho GTPase, receptor tyrosine kinase, WNT, VEGF, PI3K, and MAPK pathways is upregulated in HLF [154]. Also, the level of fibroblast growth factor 9 (FGF9) was upregulated in a rabbit model of HLF [155], and basic FGF (bFGF) expression was higher in human HLF tissues with a positive correlation with the LF thickness [156]. The available evidence failed to show the exact source of the growth factors in the LF, and how their levels are increased during the pathogenesis of HLF, but the key role of growth factors in the pathogenesis of HLF cannot be denied, and detailed attention is needed in the future to understand the pathogenesis and for the development of therapeutics against HLF. Based on these reported findings, a summarized pathway involving the growth factors is presented in Figure 2.

### 5.7. Wnt Signaling

Wnt signaling is a highly conserved pathway involved in the regulation of cell proliferation, differentiation, apoptosis, tissue homeostasis, and wound healing [157]. In a resting state, Wnt signaling is kept off, where β-catenin is phosphorylated by casein kinase 1 alpha (CK1α) and glycogen synthase kinase 3 beta (GSK3β) and other protein complexes for its degradation; and once Wnt signaling is on, GSK3β activity is inhibited leading to the stabilization of β-catenin and translocation into the nucleus where it interacts with T cell factor (TCF)/lymphoid enhancer-binding factor (LEF) transcription factors to regulate the target gene [158]. The intracellular signaling cascade activated by secreted modified proteins of the Wnt family is known as the Wnt signaling pathway, and dysregulation of this signaling leads to several diseases such as cardiac diseases, liver fibrosis, lung disorders, Parkinson’s disease, Alzheimer’s disease, osteoporosis, etc. [157,158]. Recently, studies pointed out that the Wnt signaling pathway is one of the molecular pathways involved in LF hypertrophy [159,160]. A microRNA transcriptome analysis conducted by Mori et al. showed that Wnt/β-catenin signaling along with the Aryl hydrocarbon receptor (AHR) are predicted to be associated with several miRNA signatures [160]. In another study, the LF thickness was positively correlated with age in LSS patients and showed that α-SMA, phosphorylated GSK-3β (Ser9), and β-catenin are expressed in the myofibroblasts of the dorsal layers in the LF of LSS patients [159]. Correlation analysis revealed the significant correlation between the phosphorylation of GSK-3β and the LF thickness in LSS patients [159]. A study suggested that during the fibroblast-to-myofibroblast transition, Wnt/β-catenin signaling is one of the important signaling events involved in LF hypertrophy. Studies have shown that TGFβ1 is involved in Wnt/β-catenin signaling in fibroblasts [161,162], so it is important to investigate the regulatory mechanism of GSK-3β/β-catenin in LF hypertrophy and its relationship with TGF-β.

## 6. Role of Lipid Disorders-Related Signaling in HLF

Abnormal lipid metabolism is associated with the development of various pathologies including cancer, liver diseases, cardiovascular diseases, etc. [163]. Quantitative and qualitative high-performance liquid chromatography and mass spectrometry analysis showed that the total lipid content in LF tissues from LSS patients is 3.6 times higher than that of non-hypertrophic LF tissues [164]. A study specifically identified that phosphatidylcholines (PCs), ceramides (Cers), O-acyl-ω-hydroxy fatty acids (OAHFAs), and triglycerides (TGs) were increased in HLF, with PCs and TGs showing a weak positive correlation with the LF thickness [164]. An increased level of oxidized low-density lipoprotein (LDL) is clinically involved in the pathogenesis of atherosclerosis, and in LF tissues from LSS patients, lectin-type oxidized LDL receptor 1 (LOX-1) expression is higher than that in non-LSS patients, suggesting the active LDL/LOX-1 system in HLF [165]. There was a weak but significant positive correlation between the cross-sectional area of LF and LOX-2 [165]. In LF cells, ox-LDL treatment activated the phosphorylation of ERK, p38 and JNK MAPKs, and NF-κB to induce the pro-fibrotic effects, suggesting an oxidized LDL/LOX-1 system in the pathogenesis of HLF [165]. The integrative analysis of genome-wide DNA methylation and single polymorphism analysis conducted in LF tissues identified ACSM5, a member of the medium-chain fatty acyl-CoA family, as a regulatory gene involved in the pathogenesis of HLF [55]. The methylation of ACSM5 positively correlated with the ligamentum thickness, suggesting a vital role of ACSM5 in the pathogenesis of HLF [55]. Detailed studies in LF cells showed that the hypermethylation of ACSM5 promoter by DNA-methyltransferase 1 (DNMT1) reduced the expression of ACSM5 [55,166]. The overexpression of ASCM5 in LF cells revealed an increased apoptosis, reduced proliferation, and reduced pro-fibrotic signaling indicating the protective role of ACSM5 in HLF [55]. Further, an in vivo study involving AAV-ACSM5 showed the inhibition of LF hypertrophy and lipid accumulation in a mice model of hypertrophy [166]. Mechanistically, the overexpression of ACSM5 suppressed LF hypertrophy by inhibiting the fatty acid binding protein 4 (FABP4)-mediated peroxisome proliferator-activated receptor gamma (PPARγ) signaling pathway to alleviate lipid accumulation [166]. Leptin, a hormone helping maintain normal body weight on a long-term basis, has been found to be higher in the LF from LSS patients [108]. A detailed analysis showed that leptin mRNA and protein expression had a positive correlation with the fibrosis score and LF thickness [108]. Although a direct correlation between metabolic disorders and HLF is poorly understood, the above-mentioned reports suggest that the molecular mechanisms regulating metabolic diseases are associated with HLF, making them a promising therapeutic target.

## 7. Role of Non-Coding RNAs in HLF

Non-coding RNAs (ncRNAs) are small molecules that lack the ability to encode proteins and are involved in the regulation of post-transcriptional gene expression. Micro-RNAs (miRNAs) belong to a class of small non-coding RNA molecules that include long non-coding RNAs (lncRNAs), circular RNAs (circRNAs), transfer RNAs (tRNAs), and ribosomal RNAs (rRNAs). Increasing evidence has shown the role of miRNAs in the pathogenesis of HLF. These molecules play a vital role in cellular processes such as cell differentiation, proliferation, inflammation, and apoptosis. In HLF, several ncRNAs (miR-155, miR-21, and lncRNA XIST) are upregulated [71,167,168] and many of them (miR-146a-5p, miR-221, miR-302b, miR-10396b-3p, and miR-29a) are downregulated [40,71,169,170,171,172]. Furthermore, miRNAs can rescue the fibrosis-promoting effects of lncRNAs in HLF [71].

Several miRNAs such as miR-416a-5p, miR-221-3p, miR302b-3p, miR-221, and miR-10396b-3p [71,169,170,171] have been found to possess antiproliferative properties which play a critical role in the matrix homeostasis in HLF. Notably, TIMP-2 is one of the key factors in tissue fibrosis that plays a significant role in the development of HLF. TIMP-2 promotes cell proliferation and the inhibition of apoptosis [171]. The analysis of LF fibroblast cells treated with miR-221 mimics and inhibitors demonstrate that miR-221 targets and inhibits TIMP-2 which decreases the expression of type I and III collagen and reduces HLF [171]. On the other hand, miR-10396b-3p possesses anti-inflammatory properties and targets the IL-11 pathway to inhibit fibrosis [40]. Conversely, miR-155 is pro-inflammatory and acts through the TGF-β/SMAD pathway to increase the expression of type I and III collagen [167]. The extracellular vesicles from human umbilical cord mesenchymal stem cells (hUCMSC-EVs) enriched with miR-146a-5p and miR-221-3p suppressed HLF in vivo and inhibited TGF-β1-induced fibrosis in vitro [170]. It is found that hUCMSC-EVs inhibit HLF by inhibiting TGF-β/SMAD4 signaling in LF cells [170]. LncRNA XIST, a competing endogenous RNA (ceRNA) for miR-302b-3p, is upregulated in HLF. MiR-302b-3p reverses the effects of lncRNA XIST through the regulation of VEGFA, thereby inhibiting autophagy, cell proliferation, and apoptosis in the progression of LF fibrosis [71]. These findings suggest that ncRNAs are heavily involved in the regulation and pathogenesis of HLF and that there is potential in using ncRNAs as the foundation for therapeutic strategies to delay the progression and onset of HLF. Table 1 summarizes the studies on ncRNAs in the development of HLF and fibrosis. 

## 8. In Vivo Study Model of HLF

HLF is likely a multi-factorial disease involving genetics, aging, systemic disease states, and biomechanics. Due to the complex nature of HLF, it is difficult to study the etiological and progressive aspects in humans, in which only end-stage tissue is readily available and in vivo investigation is limited to imaging modalities. Animal models are powerful tools that enable a more detailed investigation into the origins and progression of disease, providing an opportunity to conduct a greater number of investigations quickly and thoroughly while testing the effect of particular variables in the absence of confounding conditions such as aging, for example. Primary considerations are an appropriate etiological mechanism with physiologically similar disease outcomes to those observed in humans. Secondarily, one considers the species cost (husbandry), size (for surgical technique), and available tools (genetic and behavioral, among others). As a primary function of the LF is the stabilization of the spine, abnormal biomechanics are a likely cause of the hypertrophy, and recently reported models of HLF have all attempted to create spine instability to induce the disease state. The reported in vivo animal models may be organized in three categories: non-invasive instability models, mechanical overload, and surgically induced instability models.

### 8.1. Non-Invasive Instability Models

Two have been reported, both in the mouse model. In the first, abnormal biomechanics were induced through the use of a platform to which the animals were strapped and subjected to flexion/extension cycles at 3 Hz (20×/min) for 2–6 h per day [45]. The use of this specialized equipment resulted in mild hypertrophy after 6 weeks, but increased immune cell or vascular invasion, considered hallmarks of a successful model, were not observed. While the addition of a puncture wound to the LF resulted in satisfactory HLF, the etiological mechanism was disappointing. In the second, the hydrophobia of mice was used to induce bipedal posture and abnormal lumbar biomechanics [173,174]. The investigators showed that Von Mises stress and maximum principal strains within the spine were several thousand-fold higher than in the controls. HLF was observed in the experimental group, exhibiting loss of elastic fibers, increases in collagen fibers, higher cell density, and increased α-SMA positive cells as well as a higher expression of inflammatory cytokines and fibrosis-related factors such as COL1A1, COL3A1, α-SMA, MMP2, IL-1β, and COX-2 [173,174]. This model avoided any surgical manipulation to induce abnormal spine biomechanics in a model rich in genetic tools to analyze disease mechanisms. However, the husbandry, technician time, and training were considerable.

### 8.2. In Vivo Mechanical Overload Model

Hayashi et al., 2017 induced abnormal biomechanical stress at L3-4 through (1) L2-3 and L4-5 posterolateral fusion and (2) resection of the L3-4 supraspinal muscle to concentrate the mechanical stress on L3-4 in a rabbit model [155]. At 16 weeks, the authors observed LF thickening with loss of elastic fibers and an increase in collagen fibers, an increase in COL2 staining cells (indicating early calcification), and an abnormal LF and disc dimensions and mechanical properties. Further, genome-wide association studies that demonstrated a role for FGF in the pathogenesis of HLF [155] and a time course (i.e., analysis at 16 and 52 weeks) revealed clear disease progression, highlighting the emergence of α-SMA-positive cells, as shown in the mouse model [44], all indicating a pathogenic mechanism similar to the human condition. However, the rabbit is an expensive model with limited available tools for mechanistic analysis, involves a surgical procedure and instrumentation not commonly available to labs, and requires at least 16 weeks for development, i.e., substantial husbandry costs.

### 8.3. In Vivo Surgical Instability Model

A rat model of HLF was reported [175], involving the partial grinding/destruction of the facet joints, and the removal of the interspinous ligament and all the intersegmental musculature at the level of instability. Changes in spine biomechanics, LF morphology, COL and elastin fiber content, and the expression of key hypertrophic markers including TGF-β1, TNF-α, IL-1β, and COLI were reported [175]. In summary, each type of model showed LF thickening with inflammatory cell infiltration and increased COL1, COL3, and TGF-β1 production. However, none have so far advanced our understanding of the pathogenesis. This may be due to the shortcomings in the models themselves that prevent detailed analysis. On the one hand, the mechanical overload and non-invasive bipedal models require unique equipment and facilities and large time, husbandry, and resource investment that still result in variable outcomes. On the other hand, the reported surgical models are faster, less expensive, and more consistent in regard to outcome, but involve the technically difficult and highly traumatic removal of lumbar spinal pedicles and transection of numerous ligaments and muscles that render the interpretation of results difficult. 

## 9. Potential Therapeutic Strategies

Potential therapeutic targets are identified by using the well-known chemical inhibitors of the several signaling pathways involved in the pathogenesis of HLF. The therapeutic administration of cyclopamine, a steroidal alkaloid inhibitor of Hedgehog signaling, inhibited the fibrosis in a mechanical stress-induced rabbit model [56]. The treatment of rabbits with 50 mg/kg of cyclopamine every day via subcutaneous injection in a rabbit model of LF hypertrophy inhibited fibrosis-related genes at the L3-L4 level of the LF including WISP1, Gli1, COLI, and COLIII, suggesting a therapeutic potential of Hedgehog signaling inhibitor [56].

ROS scavengers are another chemical reagent having potential therapeutic potential against HLF. N-acetyl-l-cysteine (NAC) is a widely used dietary supplement and a promising agent due to its ease of administration and low toxicity. NAC has been used to treat a variety of diseases [176,177] and is used in the treatment of acetaminophen overdose in man [178]. Although there are no reports on the use of NAC for the LF in vivo, in vitro analysis pointed out the potential preventive or therapeutic effects. In vitro studies in primary human LF cells showed that NAC neutralizes the BSO- or TNF-α-induced p38 and ERK MAPK and NF-κB signaling [99]. Also, NAC inhibited the H_2_O_2_-induced inflammatory and fibrogenic markers in LF cells. NAC reversed GPx-1/2 expression, and phospho-p38 and NF-kB/p65 activation in H_2_O_2_-stimulated LF cells [176]. These studies suggest the need for more detailed studies on the therapeutic potential of antioxidants in preventing and treating HLF, as several antioxidants are already in use or are in several clinical trials [178,179,180].

Rapamycin is an autophagy-inducing agent which acts by inhibiting mTORC1. It is a clinically approved immunosuppressant agent, a well-known anti-aging agent and known for cancer prevention. It is reported that rapamycin has protective effects on human chondrocytes and disc cells [181]. In LF cells, rapamycin inhibited IGF-1-induced COLI and COLIII expression. In addition, several other studies have shown that PI3K/AKT signaling is activated in HLF (see Section 5.5), and rapamycin or other AKT/mTOR rapamycin can be promising therapeutic agents. Currently, there are no in vivo studies to report the effects of rapamycin in the prevention of HLF, so future studies are warranted. 

Rolipram, a specific phosphodiesterase (PDE) 4A and 4B inhibitor, has been known for its anti-fibrotic effects. In LF hypertrophy, the expression of PDE4A and PDE4B was significantly upregulated, with the expression of PDE4A and PDE4B showing a linear correlation [182]. In hypertrophic LF fibroblasts, rolipram inhibits COLI and TGF-β1, and inhibits TGF-β1-induced fibrotic changes in LF fibroblasts through the activation of the ERK1/2 pathway [182], suggesting rolipram as an effective anti-fibrotic agent. 

NVP-WEW541, a pyrrolo [2,3-d] pyrimidine derivative small-molecular-weight tyrosine kinase inhibitor, is known for its specific high affinity to inhibit IGF-1R and has shown promising antitumor potential in vivo. In HLF, however, a study reported its potential anti-fibrotic effects in vitro as it inhibited the IGF-1-induced AKT/mTOR pathway to inhibit COLI and COLIII expression [58], which requires further attention in the future using in vivo models. Another tyrosine kinase inhibitor, Erlotinib, is a specific EGRF inhibitor which showed an inhibitory function against EGF-induced fibrotic events in LF cells [81]. It inhibited TGF-β1, phospho-SMAD3, and COLI and COLIII in LF cells [81]. Also, the same study showed an anti-fibrotic effect of TGF-β1 neutralizing antibody in LF cells [81]. 

Simvastatin, a drug used to treat high cholesterol, reduces the expression of HLF markers such as COLI, COLIII, and COX-2 in vitro [165]. In LF cells, simvastatin inhibited the expression of pro-fibrotic markers induced by ox-LDL by inhibiting the phosphorylation of ERK, JNK, p38 MAPKs, and NF-κB [165]. 

In addition to chemical inhibitors, the genetic alteration of several signaling molecules could be effective in inhibiting fibrotic signaling in the LF. One such example is using miRNAs, as explained in Section 7, to inhibit the fibrotic signaling in the LF. Several miRNA or AAV-shRNA were delivered locally into the LF in a bipedal standing mouse model of HLF [169,170], suggesting that the local delivery of the genetic information could be utilized to treat HLF. Also, miRNA-loaded EV particles were successfully delivered to the LF in a bipedal standing mouse model of HLF, highlighting the role of EVs as bioactive materials having a promising therapy for HLF [170]. In addition, macrophage depletion in a microinjury-induced LF hypertrophy mouse model attenuated the LF hypertrophy and collagen accumulation, suggesting the potential therapeutic potential of systemic immune modulation [65]. Although most of the studies have been performed using in vitro models, few of them have used in vivo models utilizing invasive or non-invasive animal models. As explained in Section 8, the development of animal models mimicking the LSS in humans is necessary for the development and testing of therapeutics in the future. In humans, HLF is often diagnosed only after the occurrence of LSS, so future studies also should focus on the identification of certain biomarkers or early symptoms of HLF to provide early detection and treatment opportunities.

## 10. Conclusions and Future Perspectives

The growing interest in HLF as the root cause of LSS has provided a novel molecular context for its pathogenesis. Currently, there are a few effective non-surgical treatments for LSS, which means that there is a need for the development of novel non-surgical preventive or curative therapeutics. The available studies have identified aging and mechanical stress as major causes of HLF leading to LSS. These stresses further lead to the activation of inflammatory or fibrotic signaling causing the accumulation of the ECM and fibrosis of the LF. Fibroblasts are thought to be the major cell types involved, but researchers have identified other cell types such as macrophages playing a significant role in the pathogenesis of HLF. Due to the involvement of multiple cell types and cellular and molecular mechanisms, future studies should focus on identifying the crosstalk between them to pinpoint the exact molecular targets involved in the development and pathogenesis of HLF and LSS. 

Most of the studies conducted in the field of HLF have been performed using LF fibroblast cell culture models and using external cellular stressors such as TGF-β1, IL-1β, H_2_O_2_, etc., whereas very few studies have been carried out using animal models. Although several groups of researchers have developed animal models of HLF using surgical or non-surgical methods, they are still not well validated to provide the context for LSS research. Therefore, it is necessary to establish a new animal model that can accurately replicate the underlying mechanisms of HLF. This model will aid in the discovery of effective treatments for LSS, reducing the economic and health burdens associated with surgeries and their associated complications.

As the research into HLF is in its infancy, future studies are needed to identify significant therapeutic targets. Recently, omics-based studies have identified some molecular targets involved in the fibrotic signaling in the LF. However, due to the complex nature of disease progression involving multiple factors such as age and mechanical stress, and lack of understanding of how ‘LF thickening’, ‘LF fibrosis’, and ‘HLF’ are correlated, it is challenging to explore the disease pathogenesis. The development of reliable and validated animal models and a deep understanding of the molecular crosstalk between the several signaling pathways and cell types are critical to advance this research field. 

## Figures and Tables

**Table 1:** Noncoding RNAs in the hypertrophy of the ligamentum flavum.

Noncoding RNA	Known Functions	Expression in HLF	In Vitro Study Model	In Vivo Study Model	Key Findings	Ref.
miR-146a-5p miR-221-3p	Anti-proliferative, anti-inflammatory	↓	Human LF tissues and Primary mouse LF cells + TGF-β1 and hUCMSC-EVs enriched with miR-146a-5p and miR-221-3p inhibitors	Bipedal standing mice model of HLF—local injection of hUCMSC-EVs enriched with miR-146a-5p, and miR-221-3p inhibitors in LF	miRNA-enriched hUMSC-EVs could be delivered to LF hUCMSC-EVs inhibit the TGF-β/SMAD4 signaling pathway to reduce HLF	[170]
miR-221	Anti-proliferative	↓	Human primary LF cells + miR-221 mimics or inhibitors	-	miR-221 overexpression targets TIMP-2 to reduce hypertrophy by reducing the expression of COLI and COLIII in LF cells	[171]
miR-4306	Anti-proliferative, pro-apoptotic	-	Human LF tissues, primary human LF cells + miR-4306 mimics or inhibitor	Bipedal standing mice model of HLF—local injection of AAV-shTCF7 in LF	TCF7 activates SNAI2 transcription, promoting LF fibrosis and downregulates miR-4306 expression through the SNAI2/miR-4306 axis	[169]
miR-302b-3p	Anti-proliferative, pro-apoptotic	↓	Human LF samples, primary human LF cells + miR-302b-3p mimics and inhibitors	Bipedal standing mouse model of HLF—local injection of AAV-shXIST in LF	miR-302b-3p: inhibits LF cell proliferation, fibrosis, and autophagy through VEGFA *XIST:* acts as ceRNA for miR-302b-3p, promotes LF cell proliferation, fibrosis, and autophagy through XIST/miR-302b-3p/VEGFA	[71]
LncRNA XIST	Anti-apoptotic, pro-proliferative	↑				
miR-10396b-3p	Anti-inflammatory	↓	Human LF cells + IL-11, COLI, and COLIII antibodies + cyclic stretch	-	miR-10396b-3p inhibits fibrosis expression by downregulating IL-11, COLI, and COLIII through inhibiting the IL-11 pathway	[40]
miR-155	Pro-inflammatory, pro-fibrotic	↑	Human LF cells + miR-155 mimics	-	miR-155 promotes fibrosis in the LF and upregulates the expression of COLI and COLIII	[167]
miR-21	Pro-inflammatory	↑	Human LF cells + miR-21 mimics	-	miR-21 activates IL-6 to increase fibrosis and hypertrophy and elevates the expression of COLI and COLIII	[168]
miR-29a	Anti-fibrotic	↓	Human LF tissues, Primary human LF cells + miRNA 29a mimics or inhibitors	-	miR-29a regulates the collagen gene expression in the LF	[172]

Abbreviations: AAV, Adeno-associated virus; ceRNA, competing endogenous RNA; COL, collagen; hUCMSC-EVs, human umbilical cord mesenchymal stromal cell-derived extracellular vesicles; IL-6, interleukin 6; IL-11, interleukin 11; LF, ligamentum flavum; HLF, hypertrophy of ligamentum flavum; TCF7, transcription factor 7; TGF-β, transforming growth factor beta; TIMP-2, tissue inhibitor of metalloproteinases 2; SNAI2, snail family transcriptional repressor 2; VEGFA, vascular endothelial growth factor A; XIST, X inactive specific transcript; ↑, increased/higher; ↓, decreased/lower; -, not reported.

## Data Availability

Not applicable.

## References

[1] Akar E., Somay H. (2019). Comparative morphometric analysis of congenital and acquired lumbar spinal stenosis. J. Clin. Neurosci..

[2] Yoshida M., Shima K., Taniguchi Y., Tamaki T., Tanaka T. (1992). Hypertrophied ligamentum flavum in lumbar spinal canal stenosis. Pathogenesis and morphologic and immunohistochemical observation. Spine.

[3] Kolte V.S., Khambatta S., Ambiye M.V. (2015). Thickness of the ligamentum flavum: Correlation with age and its asymmetry-an magnetic resonance imaging study. Asian Spine J..

[4] Altinkaya N., Yildirim T., Demir S., Alkan O., Sarica F.B. (2011). Factors associated with the thickness of the ligamentum flavum: Is ligamentum flavum thickening due to hypertrophy or buckling?. Spine.

[5] Abbas J., Hamoud K., Masharawi Y.M., May H., Hay O., Medlej B., Peled N., Hershkovitz I. (2010). Ligamentum flavum thickness in normal and stenotic lumbar spines. Spine.

[6] Yoshiiwa T., Miyazaki M., Kawano M., Ikeda S., Tsumura H. (2016). Analysis of the Relationship between Hypertrophy of the Ligamentum Flavum and Lumbar Segmental Motion with Aging Process. Asian Spine J..

[7] Zhang K., Sun W., Liu X.Y., Zhao C.Q., Li H., Sun X.J., You-Zhuan X., Ding W., Zhao J. (2017). Hypertrophy and Fibrosis of the Ligamentum Flavum in Lumbar Spinal Stenosis is Associated With Increased Expression of LPA and LPAR1. Clin. Spine Surg..

[8] Yabe Y., Hagiwara Y., Ando A., Tsuchiya M., Minowa T., Takemura T., Honda M., Hatori K., Sonofuchi K., Kanazawa K. (2015). Chondrogenic and fibrotic process in the ligamentum flavum of patients with lumbar spinal canal stenosis. Spine.

[9] Sairyo K., Biyani A., Goel V.K., Leaman D.W., Booth R., Thomas J., Ebraheim N.A., Cowgill I.A., Mohan S.E. (2007). Lumbar ligamentum flavum hypertrophy is due to accumulation of inflammation-related scar tissue. Spine.

[10] Sairyo K., Biyani A., Goel V., Leaman D., Booth R., Thomas J., Gehling D., Vishnubhotla L., Long R., Ebraheim N. (2005). Pathomechanism of ligamentum flavum hypertrophy: A multidisciplinary investigation based on clinical, biomechanical, histologic, and biologic assessments. Spine.

[11] Hur J.W., Kim B.J., Park J.H., Kim J.H., Park Y.K., Kwon T.H., Moon H.J. (2015). The Mechanism of Ligamentum Flavum Hypertrophy: Introducing Angiogenesis as a Critical Link That Couples Mechanical Stress and Hypertrophy. Neurosurgery.

[12] Zhong Z.M., Zha D.S., Xiao W.D., Wu S.H., Wu Q., Zhang Y., Liu F.Q., Chen J.T. (2011). Hypertrophy of ligamentum flavum in lumbar spine stenosis associated with the increased expression of connective tissue growth factor. J. Orthop. Res..

[13] Pan B., Huo T., Cao M., Jing L., Luo X., Qu Z., Feng H., Yuan F., Guo K. (2021). ADAM10 promotes the proliferation of ligamentum flavum cells by activating the PI3K/AKT pathway. Int. J. Mol. Med..

[14] Chuang H.C., Tsai K.L., Tsai K.J., Tu T.Y., Shyong Y.J., Jou I.M., Hsu C.C., Shih S.S., Liu Y.F., Lin C.L. (2020). Oxidative stress mediates age-related hypertrophy of ligamentum flavum by inducing inflammation, fibrosis, and apoptosis through activating Akt and MAPK pathways. Aging.

[15] Amudong A., Muheremu A., Abudourexiti T. (2017). Hypertrophy of the ligamentum flavum and expression of transforming growth factor beta. J. Int. Med. Res..

[16] Park J.B., Chang H., Lee J.K. (2001). Quantitative analysis of transforming growth factor-beta 1 in ligamentum flavum of lumbar spinal stenosis and disc herniation. Spine.

[17] Hayashi F., Morimoto M., Higashino K., Goda Y., Sato N., Tezuka F., Yamashita K., Sairyo K. (2022). Myofibroblasts are increased in the dorsal layer of the hypertrophic ligamentum flavum in lumbar spinal canal stenosis. Spine J..

[18] Salimi H., Suzuki A., Habibi H., Orita K., Hori Y., Yabu A., Terai H., Tamai K., Nakamura H. (2021). Biglycan expression and its function in human ligamentum flavum. Sci. Rep..

[19] Khalil H., Kanisicak O., Prasad V., Correll R.N., Fu X., Schips T., Vagnozzi R.J., Liu R., Huynh T., Lee S.J. (2017). Fibroblast-specific TGF-beta-Smad2/3 signaling underlies cardiac fibrosis. J. Clin. Investig..

[20] Zhang X., Ma Y., You T., Tian X., Zhang H., Zhu Q., Zhang W. (2015). Roles of TGF-beta/Smad signaling pathway in pathogenesis and development of gluteal muscle contracture. Connect. Tissue Res..

[21] Wrana J.L., Attisano L. (2000). The Smad pathway. Cytokine Growth Factor. Rev..

[22] George K.M., Hernandez N.S., Breton J., Cooper B., Dowd R.S., Nail J., Yu A., Mastroianni M., Wang A., Godara A. (2021). Lumbar ligamentum flavum burden: Evaluating the role of ATTRwt amyloid deposition in ligamentum flavum thickness at all lumbar levels. Clin. Neurol. Neurosurg..

[23] Buchbinder R., van Tulder M., Oberg B., Costa L.M., Woolf A., Schoene M., Croft P., Lancet Low Back Pain Series Working G. (2018). Low back pain: A call for action. Lancet.

[24] Deyo R.A., Mirza S.K., Martin B.I., Kreuter W., Goodman D.C., Jarvik J.G. (2010). Trends, major medical complications, and charges associated with surgery for lumbar spinal stenosis in older adults. JAMA.

[25] Katz J.N., Zimmerman Z.E., Mass H., Makhni M.C. (2022). Diagnosis and Management of Lumbar Spinal Stenosis: A Review. JAMA.

[26] Ravindra V.M., Senglaub S.S., Rattani A., Dewan M.C., Hartl R., Bisson E., Park K.B., Shrime M.G. (2018). Degenerative Lumbar Spine Disease: Estimating Global Incidence and Worldwide Volume. Global Spine J..

[27] Lurie J., Tomkins-Lane C. (2016). Management of lumbar spinal stenosis. BMJ.

[28] Deyo R.A. (2010). Treatment of lumbar spinal stenosis: A balancing act. Spine J..

[29] Deer T., Sayed D., Michels J., Josephson Y., Li S., Calodney A.K. (2019). A Review of Lumbar Spinal Stenosis with Intermittent Neurogenic Claudication: Disease and Diagnosis. Pain Med..

[30] Raad M., Donaldson C.J., El Dafrawy M.H., Sciubba D.M., Riley L.H., Neuman B.J., Kebaish K.M., Skolasky R.L. (2018). Trends in isolated lumbar spinal stenosis surgery among working US adults aged 40–64 years, 2010–2014. J. Neurosurg. Spine.

[31] Weinstein J.N., Tosteson T.D., Lurie J.D., Tosteson A., Blood E., Herkowitz H., Cammisa F., Albert T., Boden S.D., Hilibrand A. (2010). Surgical versus nonoperative treatment for lumbar spinal stenosis four-year results of the Spine Patient Outcomes Research Trial. Spine.

[32] Ammendolia C., Stuber K., de Bruin L.K., Furlan A.D., Kennedy C.A., Rampersaud Y.R., Steenstra I.A., Pennick V. (2012). Nonoperative treatment of lumbar spinal stenosis with neurogenic claudication: A systematic review. Spine.

[33] Deer T.R., Grider J.S., Pope J.E., Lamer T.J., Wahezi S.E., Hagedorn J.M., Falowski S., Tolba R., Shah J.M., Strand N. (2022). Best Practices for Minimally Invasive Lumbar Spinal Stenosis Treatment 2.0 (MIST): Consensus Guidance from the American Society of Pain and Neuroscience (ASPN). J. Pain. Res..

[34] Porter R.W. (1996). Spinal stenosis and neurogenic claudication. Spine.

[35] Beamer Y.B., Garner J.T., Shelden C.H. (1973). Hypertrophied ligamentum flavum. Clinical and surgical significance. Arch. Surg..

[36] Sakamaki T., Sairyo K., Sakai T., Tamura T., Okada Y., Mikami H. (2009). Measurements of ligamentum flavum thickening at lumbar spine using MRI. Arch. Orthop. Trauma. Surg..

[37] Munns J.J., Lee J.Y., Espinoza Orias A.A., Takatori R., Andersson G.B., An H.S., Inoue N. (2015). Ligamentum flavum hypertrophy in asymptomatic and chronic low back pain subjects. PLoS ONE.

[38] Safak A.A., Is M., Sevinc O., Barut C., Eryoruk N., Erdogmus B., Dosoglu M. (2010). The thickness of the ligamentum flavum in relation to age and gender. Clin. Anat..

[39] Nakatani T., Marui T., Hitora T., Doita M., Nishida K., Kurosaka M. (2002). Mechanical stretching force promotes collagen synthesis by cultured cells from human ligamentum flavum via transforming growth factor-beta1. J. Orthop. Res..

[40] Li P., Liu C., Qian L., Zheng Z., Li C., Lian Z., Liu J., Zhang Z., Wang L. (2021). miR-10396b-3p inhibits mechanical stress-induced ligamentum flavum hypertrophy by targeting IL-11. FASEB J..

[41] Liu C., Li P., Ao X., Lian Z., Liu J., Li C., Huang M., Wang L., Zhang Z. (2022). Clusterin negatively modulates mechanical stress-mediated ligamentum flavum hypertrophy through TGF-beta1 signaling. Exp. Mol. Med..

[42] Chen J., Liu Z., Zhong G., Li Z., Qian L., Li X., Chen B., Lao L., Wang H. (2016). Cyclic stretch enhances apoptosis in human lumbar ligamentum fl avum cells via the induction of reactive oxygen species generation. J. Spinal Cord. Med..

[43] Kwon W.K., Ham C.H., Choi H., Baek S.M., Lee J.W., Park Y.K., Moon H.J., Park W.B., Kim J.H. (2022). Elucidating the effect of mechanical stretch stress on the mechanism of ligamentum flavum hypertrophy: Development of a novel in vitro multi-torsional stretch loading device. PLoS ONE.

[44] Hori Y., Suzuki A., Hayashi K., Ohyama S., Yabu A., Maruf M.H., Habibi H., Salimi H., Nakamura H. (2021). Long-term, Time-course Evaluation of Ligamentum Flavum Hypertrophy Induced by Mechanical Stress: An Experimental Animal Study. Spine.

[45] Saito T., Yokota K., Kobayakawa K., Hara M., Kubota K., Harimaya K., Kawaguchi K., Hayashida M., Matsumoto Y., Doi T. (2017). Experimental Mouse Model of Lumbar Ligamentum Flavum Hypertrophy. PLoS ONE.

[46] Diller R.B., Tabor A.J. (2022). The Role of the Extracellular Matrix (ECM) in Wound Healing: A Review. Biomimetics.

[47] Wight T.N., Potter-Perigo S. (2011). The extracellular matrix: An active or passive player in fibrosis?. Am. J. Physiol. Gastrointest. Liver Physiol..

[48] Klingberg F., Hinz B., White E.S. (2013). The myofibroblast matrix: Implications for tissue repair and fibrosis. J. Pathol..

[49] Lee S.B., Kalluri R. (2010). Mechanistic connection between inflammation and fibrosis. Kidney Int. Suppl..

[50] Ueshima E., Fujimori M., Kodama H., Felsen D., Chen J., Durack J.C., Solomon S.B., Coleman J.A., Srimathveeravalli G. (2019). Macrophage-secreted TGF-beta(1) contributes to fibroblast activation and ureteral stricture after ablation injury. Am. J. Physiol. Renal Physiol..

[51] Frangogiannis N. (2020). Transforming growth factor-beta in tissue fibrosis. J. Exp. Med..

[52] Hur J.W., Bae T., Ye S., Kim J.H., Lee S., Kim K., Lee S.H., Kim J.S., Lee J.B., Cho T.H. (2017). Myofibroblast in the ligamentum flavum hypertrophic activity. Eur. Spine J..

[53] Desmouliere A., Badid C., Bochaton-Piallat M.L., Gabbiani G. (1997). Apoptosis during wound healing, fibrocontractive diseases and vascular wall injury. Int. J. Biochem. Cell Biol..

[54] Wilkinson H.N., Hardman M.J. (2020). Wound healing: Cellular mechanisms and pathological outcomes. Open Biol..

[55] Cao Y., Zhan Y., Qiu S., Chen Z., Gong K., Ni S., Duan Y. (2021). Integrative analysis of genome-wide DNA methylation and single-nucleotide polymorphism identified ACSM5 as a suppressor of lumbar ligamentum flavum hypertrophy. Arthritis Res. Ther..

[56] Sun C., Ma Q., Yin J., Zhang H., Liu X. (2021). WISP-1 induced by mechanical stress contributes to fibrosis and hypertrophy of the ligamentum flavum through Hedgehog-Gli1 signaling. Exp. Mol. Med..

[57] Zhou T., Du L., Chen C., Han C., Li X., Qin A., Zhao C., Zhang K., Zhao J. (2018). Lysophosphatidic Acid Induces Ligamentum Flavum Hypertrophy Through the LPAR1/Akt Pathway. Cell. Physiol. Biochem..

[58] Yan B., Huang M., Zeng C., Yao N., Zhang J., Yan B., Jiang H., Tian X., Ao X., Zhao H. (2018). Locally Produced IGF-1 Promotes Hypertrophy of the Ligamentum Flavum via the mTORC1 Signaling Pathway. Cell. Physiol. Biochem..

[59] Lu Q.L., Zheng Z.X., Ye Y.H., Lu J.Y., Zhong Y.Q., Sun C., Xiong C.J., Yang G.X., Xu F. (2022). Macrophage migration inhibitory factor takes part in the lumbar ligamentum flavum hypertrophy. Mol. Med. Rep..

[60] Yang H., Cheng H., Dai R., Shang L., Zhang X., Wen H. (2023). Macrophage polarization in tissue fibrosis. PeerJ.

[61] Wynn T.A., Vannella K.M. (2016). Macrophages in Tissue Repair, Regeneration, and Fibrosis. Immunity.

[62] Patel N.K., Nunez J.H., Sorkin M., Marini S., Pagani C.A., Strong A.L., Hwang C.D., Li S., Padmanabhan K.R., Kumar R. (2022). Macrophage TGF-beta signaling is critical for wound healing with heterotopic ossification after trauma. JCI Insight.

[63] Liu Z., Kuang W., Zhou Q., Zhang Y. (2018). TGF-beta1 secreted by M2 phenotype macrophages enhances the stemness and migration of glioma cells via the SMAD2/3 signalling pathway. Int. J. Mol. Med..

[64] Murray L.A., Chen Q., Kramer M.S., Hesson D.P., Argentieri R.L., Peng X., Gulati M., Homer R.J., Russell T., van Rooijen N. (2011). TGF-beta driven lung fibrosis is macrophage dependent and blocked by *Serum amyloid* P. Int. J. Biochem. Cell Biol..

[65] Saito T., Hara M., Kumamaru H., Kobayakawa K., Yokota K., Kijima K., Yoshizaki S., Harimaya K., Matsumoto Y., Kawaguchi K. (2017). Macrophage Infiltration Is a Causative Factor for Ligamentum Flavum Hypertrophy through the Activation of Collagen Production in Fibroblasts. Am. J. Pathol..

[66] Yang J., Chen G., Fan T., Qu X. (2024). M1 macrophage-derived oncostatin M induces osteogenic differentiation of ligamentum flavum cells through the JAK2/STAT3 pathway. JOR Spine.

[67] Qu X., Xu G., Hou X., Chen G., Fan T., Yang X., Chen Z. (2022). M1 Macrophage-Derived Interleukin-6 Promotes the Osteogenic Differentiation of Ligamentum Flavum Cells. Spine.

[68] Hill C., Wang Y. (2022). Autophagy in pulmonary fibrosis: Friend or foe?. Genes. Dis..

[69] Liang S., Wu Y.S., Li D.Y., Tang J.X., Liu H.F. (2022). Autophagy and Renal Fibrosis. Aging Dis..

[70] Takagaki Y., Lee S.M., Dongqing Z., Kitada M., Kanasaki K., Koya D. (2020). Endothelial autophagy deficiency induces IL6—Dependent endothelial mesenchymal transition and organ fibrosis. Autophagy.

[71] Cao Y., Li J., Qiu S., Ni S., Duan Y. (2023). LncRNA XIST facilitates hypertrophy of ligamentum flavum by activating VEGFA-mediated autophagy through sponging miR-302b-3p. Biol. Direct.

[72] Chen J., Yu X., Qiu M., Feng F., Liu Z., Zhong G. (2021). Circular RNA Expression Profile in Patients with Lumbar Spinal Stenosis Associated with Hypertrophied Ligamentum Flavum. Spine.

[73] Li P., Fei C.S., Chen Y.L., Chen Z.S., Lai Z.M., Tan R.Q., Yu Y.P., Xiang X., Dong J.L., Zhang J.X. (2022). Revealing the novel autophagy-related genes for ligamentum flavum hypertrophy in patients and mice model. Front. Immunol..

[74] Dai R., Zhang L., Jin H., Wang D., Cheng M., Sang T., Peng C., Li Y., Wang Y. (2022). Autophagy in renal fibrosis: Protection or promotion?. Front. Pharmacol..

[75] Massague J., Sheppard D. (2023). TGF-beta signaling in health and disease. Cell.

[76] Biernacka A., Dobaczewski M., Frangogiannis N.G. (2011). TGF-beta signaling in fibrosis. Growth Factors.

[77] Chen P.Y., Qin L., Simons M. (2023). TGFbeta signaling pathways in human health and disease. Front. Mol. Biosci..

[78] Hata A., Chen Y.G. (2016). TGF-beta Signaling from Receptors to Smads. Cold Spring Harb. Perspect. Biol..

[79] Derynck R., Zhang Y.E. (2003). Smad-dependent and Smad-independent pathways in TGF-beta family signalling. Nature.

[80] Prud’homme G.J. (2007). Pathobiology of transforming growth factor beta in cancer, fibrosis and immunologic disease, and therapeutic considerations. Lab. Investig..

[81] Yang K., Chen Y., Xiang X., Lin Y., Fei C., Chen Z., Lai Z., Yu Y., Tan R., Dong J. (2022). EGF Contributes to Hypertrophy of Human Ligamentum Flavum via the TGF-beta1/Smad3 Signaling Pathway. Int. J. Med. Sci..

[82] Lohr M., Hampl J.A., Lee J.Y., Ernestus R.I., Deckert M., Stenzel W. (2011). Hypertrophy of the lumbar ligamentum flavum is associated with inflammation-related TGF-beta expression. Acta Neurochir..

[83] Schrader P.K., Grob D., Rahn B.A., Cordey J., Dvorak J. (1999). Histology of the ligamentum flavum in patients with degenerative lumbar spinal stenosis. Eur. Spine J..

[84] Kim H.J., Park J.B., Won H.Y., Chang H. (2007). Serum Levels of TGF-beta1, TIMP-1 and TIMP-2 in Patients with Lumbar Spinal Stenosis and Disc Herniation. Asian Spine J..

[85] Wynn T.A., Barron L. (2010). Macrophages: Master regulators of inflammation and fibrosis. Semin. Liver Dis..

[86] Shi X., Young C.D., Zhou H., Wang X. (2020). Transforming Growth Factor-beta Signaling in Fibrotic Diseases and Cancer-Associated Fibroblasts. Biomolecules.

[87] Wang L., Chang M., Tian Y., Yan J., Xu W., Yuan S., Zhang K., Liu X. (2021). The Role of Smad2 in Transforming Growth Factor beta(1)-Induced Hypertrophy of Ligamentum Flavum. World Neurosurg..

[88] Cao Y.L., Duan Y., Zhu L.X., Zhan Y.N., Min S.X., Jin A.M. (2016). TGF-beta1, in association with the increased expression of connective tissue growth factor, induce the hypertrophy of the ligamentum flavum through the p38 MAPK pathway. Int. J. Mol. Med..

[89] Ye S., Kwon W.K., Bae T., Kim S., Lee J.B., Cho T.H., Park J.Y., Kim K., Hur J.K., Hur J.W. (2019). CCN5 Reduces Ligamentum Flavum Hypertrophy by Modulating the TGF-beta Pathway. J. Orthop. Res..

[90] Steele H., Cheng J., Willicut A., Dell G., Breckenridge J., Culberson E., Ghastine A., Tardif V., Herro R. (2023). TNF superfamily control of tissue remodeling and fibrosis. Front. Immunol..

[91] Denga T.M., Gunter S., Fourie S., Roux R.L., Manilall A., Millen A.M.E., Mokotedi L. (2023). Interleukin-6 Blockers Improve Inflammation-Induced Lipid Metabolism Impairments but Induce Liver Fibrosis in Collagen-Induced Arthritis. Endocr. Metab. Immune Disord. Drug Targets.

[92] Tanaka H., Sun T., Kinashi H., Kamiya K., Yamaguchi M., Nobata H., Sakata F., Kim H., Mizuno M., Kunoki S. (2022). Interleukin-6 blockade reduces salt-induced cardiac inflammation and fibrosis in subtotal nephrectomized mice. Am. J. Physiol. Renal Physiol..

[93] Chen W., Yuan H., Cao W., Wang T., Chen W., Yu H., Fu Y., Jiang B., Zhou H., Guo H. (2019). Blocking interleukin-6 trans-signaling protects against renal fibrosis by suppressing STAT3 activation. Theranostics.

[94] Rose-John S., Winthrop K., Calabrese L. (2017). The role of IL-6 in host defence against infections: Immunobiology and clinical implications. Nat. Rev. Rheumatol..

[95] Borthwick L.A. (2016). The IL-1 cytokine family and its role in inflammation and fibrosis in the lung. Semin. Immunopathol..

[96] Tylutka A., Walas L., Zembron-Lacny A. (2024). Level of IL-6, TNF, and IL-1beta and age-related diseases: A systematic review and meta-analysis. Front. Immunol..

[97] Chen X., Wang Z., Deng R., Yan H., Liu X., Kang R. (2023). Intervertebral disc degeneration and inflammatory microenvironment: Expression, pathology, and therapeutic strategies. Inflamm. Res..

[98] Risbud M.V., Shapiro I.M. (2014). Role of cytokines in intervertebral disc degeneration: Pain and disc content. Nat. Rev. Rheumatol..

[99] Ito K., Kise H., Suzuki S., Nagai S., Hachiya K., Takeda H., Kawabata S., Ikeda D., Takubo K., Kaneko S. (2023). Potential Involvement of Oxidative Stress in Ligamentum Flavum Hypertrophy. J. Clin. Med..

[100] Yagi K., Goto Y., Kato K., Suzuki N., Kondo A., Waseda Y., Mizutani J., Kawaguchi Y., Joyo Y., Waguri-Nagaya Y. (2021). p38 Mitogen-Activated Protein Kinase Is Involved in Interleukin-6 Secretion from Human Ligamentum Flavum-Derived Cells Stimulated by Tumor Necrosis Factor-alpha. Asian Spine J..

[101] Zheng Z., Ao X., Li P., Lian Z., Jiang T., Zhang Z., Wang L. (2020). CRLF1 Is a Key Regulator in the Ligamentum Flavum Hypertrophy. Front. Cell Dev. Biol..

[102] Kim B.J., Hur J.W., Park J.S., Kim J.H., Kwon T.H., Park Y.K., Moon H.J. (2016). Expression of matrix metalloproteinase-2 and -9 in human ligamentum flavum cells treated with tumor necrosis factor-alpha and interleukin-1beta. J. Neurosurg. Spine.

[103] Hsu Y.H., Chen C.N., Chang H.I., Tsai H.L., Chang Y.H., Cheng I.S., Yang Y.S., Huang K.Y. (2023). Manipulation of osteogenic and adipogenic differentiation of human degenerative disc and ligamentum flavum derived progenitor cells using IL-1beta, IL-19, and IL-20. Eur. Spine J..

[104] Goto Y., Kato K., Yagi K., Kawaguchi Y., Yonezu H., Koshimae T., Waguri-Nagaya Y., Murakami H., Suzuki N. (2023). Transforming Growth Factor-beta Induces Interleukin-6 Secretion from Human Ligamentum Flavum-Derived Cells through Partial Activation of p38 and p44/42 Mitogen-Activated Protein Kinases. Asian Spine J..

[105] Nakamura T., Okada T., Endo M., Nakamura T., Oike Y., Mizuta H. (2015). Angiopoietin-like protein 2 promotes inflammatory conditions in the ligamentum flavum in the pathogenesis of lumbar spinal canal stenosis by activating interleukin-6 expression. Eur. Spine J..

[106] Yamahata H., Osuka K., Aoyama T., Yasuda M., Tokimura H., Arita K., Takayasu M. (2017). Expression of the JAK/STAT signaling pathway in the ligamentum flavum of patients with lumbar spinal canal stenosis. J. Orthop. Sci..

[107] Yabu A., Suzuki A., Hayashi K., Hori Y., Terai H., Orita K., Habibi H., Salimi H., Kono H., Toyoda H. (2023). Periostin increased by mechanical stress upregulates interleukin-6 expression in the ligamentum flavum. FASEB J..

[108] Sun C., Wang Z., Tian J.W., Wang Y.H. (2018). Leptin-induced inflammation by activating IL-6 expression contributes to the fibrosis and hypertrophy of ligamentum flavum in lumbar spinal canal stenosis. Biosci. Rep..

[109] Crisponi L., Buers I., Rutsch F. (2022). CRLF1 and CLCF1 in Development, Health and Disease. Int. J. Mol. Sci..

[110] Pizzino G., Irrera N., Cucinotta M., Pallio G., Mannino F., Arcoraci V., Squadrito F., Altavilla D., Bitto A. (2017). Oxidative Stress: Harms and Benefits for Human Health. Oxid. Med. Cell Longev..

[111] Finkel T., Holbrook N.J. (2000). Oxidants, oxidative stress and the biology of ageing. Nature.

[112] Estornut C., Milara J., Bayarri M.A., Belhadj N., Cortijo J. (2021). Targeting Oxidative Stress as a Therapeutic Approach for Idiopathic Pulmonary Fibrosis. Front. Pharmacol..

[113] Roehlen N., Crouchet E., Baumert T.F. (2020). Liver Fibrosis: Mechanistic Concepts and Therapeutic Perspectives. Cells.

[114] Otoupalova E., Smith S., Cheng G., Thannickal V.J. (2020). Oxidative Stress in Pulmonary Fibrosis. Compr. Physiol..

[115] Su H., Wan C., Song A., Qiu Y., Xiong W., Zhang C. (2019). Oxidative Stress and Renal Fibrosis: Mechanisms and Therapies. Adv. Exp. Med. Biol..

[116] Richter K., Kietzmann T. (2016). Reactive oxygen species and fibrosis: Further evidence of a significant liaison. Cell Tissue Res..

[117] Richter K., Konzack A., Pihlajaniemi T., Heljasvaara R., Kietzmann T. (2015). Redox-fibrosis: Impact of TGFbeta1 on ROS generators, mediators and functional consequences. Redox Biol..

[118] Gu Y., Yu W., Qi M., Hu J., Jin Q., Wang X., Wang C., Chen Y., Yuan W. (2023). Identification and validation of hub genes and pathways associated with mitochondrial dysfunction in hypertrophy of ligamentum flavum. Front. Genet..

[119] Palma F.R., Gantner B.N., Sakiyama M.J., Kayzuka C., Shukla S., Lacchini R., Cunniff B., Bonini M.G. (2024). ROS production by mitochondria: Function or dysfunction?. Oncogene.

[120] Dechsupa S., Yingsakmongkol W., Limthongkul W., Singhatanadgige W., Honsawek S. (2018). Relative telomere length and oxidative DNA damage in hypertrophic ligamentum flavum of lumbar spinal stenosis. PeerJ.

[121] Yucetas S.C., Cakir T. (2019). Decreased catalase expression is associated with ligamentum flavum hypertrophy due to lumbar spinal canal stenosis. Medicine.

[122] Hybertson B.M., Gao B., Bose S.K., McCord J.M. (2011). Oxidative stress in health and disease: The therapeutic potential of Nrf2 activation. Mol. Aspects Med..

[123] Marchev A.S., Dimitrova P.A., Burns A.J., Kostov R.V., Dinkova-Kostova A.T., Georgiev M.I. (2017). Oxidative stress and chronic inflammation in osteoarthritis: Can NRF2 counteract these partners in crime?. Ann. N. Y. Acad. Sci..

[124] Gu Y., Hu J., Wang C., Qi M., Chen Y., Yu W., Wang Z., Wang X., Yuan W. (2023). Smurf1 Facilitates Oxidative Stress and Fibrosis of Ligamentum Flavum by Promoting Nrf2 Ubiquitination and Degradation. Mediat. Inflamm..

[125] Merline R., Schaefer R.M., Schaefer L. (2009). The matricellular functions of small leucine-rich proteoglycans (SLRPs). J. Cell Commun. Signal.

[126] Wang S., Qu Y., Fang X., Ding Q., Zhao H., Yu X., Xu T., Lu R., Jing S., Liu C. (2023). Decorin: A potential therapeutic candidate for ligamentum flavum hypertrophy by antagonizing TGF-beta1. Exp. Mol. Med..

[127] Zhao R., Dong J., Liu C., Li M., Tan R., Fei C., Chen Y., Yang X., Shi J., Xu J. (2024). Thrombospondin-1 promotes mechanical stress-mediated ligamentum flavum hypertrophy through the TGFbeta1/Smad3 signaling pathway. Matrix Biol..

[128] Yeger H., Perbal B. (2007). The CCN family of genes: A perspective on CCN biology and therapeutic potential. J. Cell Commun. Signal.

[129] Sun C., Zhang H., Liu X. (2021). Emerging role of CCN family proteins in fibrosis. J. Cell Physiol..

[130] Lipson K.E., Wong C., Teng Y., Spong S. (2012). CTGF is a central mediator of tissue remodeling and fibrosis and its inhibition can reverse the process of fibrosis. Fibrogenesis Tissue Repair.

[131] Sun C., Liu X., Guan G., Zhang H. (2017). Increased expression of WISP-1 (CCN4) contributes to fibrosis in the hypertrophied lumber ligamentum flavum. Int. J. Clin. Exp. Pathol..

[132] Jeong D., Lee M.A., Li Y., Yang D.K., Kho C., Oh J.G., Hong G., Lee A., Song M.H., LaRocca T.J. (2016). Matricellular Protein CCN5 Reverses Established Cardiac Fibrosis. J. Am. Coll. Cardiol..

[133] Widmann C., Gibson S., Jarpe M.B., Johnson G.L. (1999). Mitogen-activated protein kinase: Conservation of a three-kinase module from yeast to human. Physiol. Rev..

[134] Strand D.W., Liang Y.Y., Yang F., Barron D.A., Ressler S.J., Schauer I.G., Feng X.H., Rowley D.R. (2014). TGF-beta induction of FGF-2 expression in stromal cells requires integrated smad3 and MAPK pathways. Am. J. Clin. Exp. Urol..

[135] Zhang W., Liu H.T. (2002). MAPK signal pathways in the regulation of cell proliferation in mammalian cells. Cell Res..

[136] Chao Y.H., Tsuang Y.H., Sun J.S., Sun M.G., Chen M.H. (2012). Centrifugal force induces human ligamentum flavum fibroblasts inflammation through activation of JNK and p38 pathways. Connect. Tissue Res..

[137] Sieber P., Schafer A., Lieberherr R., Caimi S.L., Luthi U., Ryge J., Bergmann J.H., Le Goff F., Stritt M., Blattmann P. (2023). NF-kappaB drives epithelial-mesenchymal mechanisms of lung fibrosis in a translational lung cell model. JCI Insight.

[138] Yu H., Lin L., Zhang Z., Zhang H., Hu H. (2020). Targeting NF-kappaB pathway for the therapy of diseases: Mechanism and clinical study. Signal Transduct. Target. Ther..

[139] Lawrence T. (2009). The nuclear factor NF-kappaB pathway in inflammation. Cold Spring Harb. Perspect. Biol..

[140] Jin G., Su Y., Dong Q., Zhao X., Zhang L., Yan X. (2019). Arctigenin alleviates TGF-beta1-induced epithelial-mesenchymal transition and PAI-1 expression via AMPK/NF-kappaB pathway in peritoneal mesothelial cells. Biochem. Biophys. Res. Commun..

[141] Luo K. (2017). Signaling Cross Talk between TGF-beta/Smad and Other Signaling Pathways. Cold Spring Harb. Perspect. Biol..

[142] Freudlsperger C., Bian Y., Contag Wise S., Burnett J., Coupar J., Yang X., Chen Z., Van Waes C. (2013). TGF-beta and NF-kappaB signal pathway cross-talk is mediated through TAK1 and SMAD7 in a subset of head and neck cancers. Oncogene.

[143] Ersahin T., Tuncbag N., Cetin-Atalay R. (2015). The PI3K/AKT/mTOR interactive pathway. Mol. Biosyst..

[144] Duan Y., Ni S., Zhao K., Qian J., Hu X. (2022). Immune cell infiltration and the genes associated with ligamentum flavum hypertrophy: Identification and validation. Front. Cell Dev. Biol..

[145] Yan B., Zeng C., Chen Y., Huang M., Yao N., Zhang J., Yan B., Tang J., Wang L., Zhang Z. (2021). Mechanical Stress-Induced IGF-1 Facilitates col-I and col-III Synthesis via the IGF-1R/AKT/mTORC1 Signaling Pathway. Stem Cells Int..

[146] National Cancer Institute Clinical Trials Using Akt Inhibitor. https://www.cancer.gov/research/participate/clinical-trials/intervention/akt-inhibitor.

[147] Desai T.J., Cardoso W.V. (2002). Growth factors in lung development and disease: Friends or foe?. Respir. Res..

[148] Jirathanathornnukul N., Limthongkul W., Yingsakmongkol W., Singhatanadgige W., Parkpian V., Honsawek S. (2016). Increased expression of vascular endothelial growth factor is associated with hypertrophic ligamentum flavum in lumbar spinal canal stenosis. J. Investig. Med..

[149] Martin R., Gutierrez B., Cordova C., Roman A.S., Alvarez Y., Hernandez M., Cachofeiro V., Nieto M.L. (2020). Secreted Phospholipase A(2)-IIA Modulates Transdifferentiation of Cardiac Fibroblast through EGFR Transactivation: An Inflammation-Fibrosis Link. Cells.

[150] Ingram J.L., Bonner J.C. (2006). EGF and PDGF receptor tyrosine kinases as therapeutic targets for chronic lung diseases. Curr. Mol. Med..

[151] Komuves L.G., Feren A., Jones A.L., Fodor E. (2000). Expression of epidermal growth factor and its receptor in cirrhotic liver disease. J. Histochem. Cytochem..

[152] Cheng W.H., Kao S.Y., Chen C.L., Yuliani F.S., Lin L.Y., Lin C.H., Chen B.C. (2022). Amphiregulin induces CCN2 and fibronectin expression by TGF-beta through EGFR-dependent pathway in lung epithelial cells. Respir. Res..

[153] Zhao Y., Ma J., Fan Y., Wang Z., Tian R., Ji W., Zhang F., Niu R. (2018). TGF-beta transactivates EGFR and facilitates breast cancer migration and invasion through canonical Smad3 and ERK/Sp1 signaling pathways. Mol. Oncol..

[154] Guevara-Villazon F., Pacheco-Tena C., Anchondo-Lopez A., Ordonez-Solorio L.A., Contreras Martinez B., Munoz-Cobos A., Luevano-Gonzalez A., Gonzalez-Chavez S.A. (2023). Transcriptomic alterations in hypertrophy of the ligamentum flavum: Interactions of Rho GTPases, RTK, PIK3, and FGF. Eur. Spine J..

[155] Hayashi K., Suzuki A., Terai H., Ahmadi S.A., Rahmani M.S., Maruf M.H., Habibi H., Hori Y., Yamada K., Hoshino M. (2019). Fibroblast Growth Factor 9 Is Upregulated Upon Intervertebral Mechanical Stress-Induced Ligamentum Flavum Hypertrophy in a Rabbit Model. Spine.

[156] Honsawek S., Poonpukdee J., Chalermpanpipat C., Payungporn S., Limthongkul W., Yingsakmongkol W., Thanakit V., Parkpian V. (2013). Hypertrophy of the ligamentum flavum in lumbar spinal canal stenosis is associated with increased bFGF expression. Int. Orthop..

[157] Liu J., Xiao Q., Xiao J., Niu C., Li Y., Zhang X., Zhou Z., Shu G., Yin G. (2022). Wnt/beta-catenin signalling: Function, biological mechanisms, and therapeutic opportunities. Signal Transduct. Target. Ther..

[158] Rim E.Y., Clevers H., Nusse R. (2022). The Wnt Pathway: From Signaling Mechanisms to Synthetic Modulators. Annu. Rev. Biochem..

[159] Shin H.K., Seo K.J., Lee J.Y., Jeon S.R., Yune T.Y. (2023). GSK-3beta and beta-Catenin Signaling Pathway is Involved in Myofibroblast Transition of Ligamentum Flavum in Lumbar Spinal Stenosis Patients. Spine.

[160] Mori T., Sakai Y., Kayano M., Matsuda A., Oboki K., Matsumoto K., Harada A., Niida S., Watanabe K. (2017). MicroRNA transcriptome analysis on hypertrophy of ligamentum flavum in patients with lumbar spinal stenosis. Spine Surg. Relat. Res..

[161] Vallee A., Lecarpentier Y., Guillevin R., Vallee J.N. (2017). Interactions between TGF-beta1, canonical WNT/beta-catenin pathway and PPAR gamma in radiation-induced fibrosis. Oncotarget.

[162] Caraci F., Gili E., Calafiore M., Failla M., La Rosa C., Crimi N., Sortino M.A., Nicoletti F., Copani A., Vancheri C. (2008). TGF-beta1 targets the GSK-3beta/beta-catenin pathway via ERK activation in the transition of human lung fibroblasts into myofibroblasts. Pharmacol. Res..

[163] Natesan V., Kim S.J. (2021). Lipid Metabolism, Disorders and Therapeutic Drugs—Review. Biomol. Ther..

[164] Yamada T., Horikawa M., Sato T., Kahyo T., Takanashi Y., Ushirozako H., Kurosu K., Al Mamun M., Mihara Y., Oe S. (2021). Hypertrophy of the ligamentum flavum in lumbar spinal canal stenosis is associated with abnormal accumulation of specific lipids. Sci. Rep..

[165] Nagai S., Hachiya K., Takeda H., Ikeda D., Kawabata S., Watanabe K., Kaneko S., Fujita N. (2023). Impact of oxidized LDL/LOX-1 system on ligamentum flavum hypertrophy. J. Orthop. Sci..

[166] Cao Y., Li J., Qiu S., Ni S., Duan Y. (2023). ACSM5 inhibits ligamentum flavum hypertrophy by regulating lipid accumulation mediated by FABP4/PPAR signaling pathway. Biol. Direct.

[167] Chen J., Liu Z., Zhong G., Qian L., Li Z., Qiao Z., Chen B., Wang H. (2014). Hypertrophy of ligamentum flavum in lumbar spine stenosis is associated with increased miR-155 level. Dis. Markers.

[168] Sun C., Tian J., Liu X., Guan G. (2017). MiR-21 promotes fibrosis and hypertrophy of ligamentum flavum in lumbar spinal canal stenosis by activating IL-6 expression. Biochem. Biophys. Res. Commun..

[169] Duan Y., Li J., Qiu S., Ni S., Cao Y. (2022). TCF7/SNAI2/miR-4306 feedback loop promotes hypertrophy of ligamentum flavum. J. Transl. Med..

[170] Ma C., Qi X., Wei Y.F., Li Z., Zhang H.L., Li H., Yu F.L., Pu Y.N., Huang Y.C., Ren Y.X. (2023). Amelioration of ligamentum flavum hypertrophy using umbilical cord mesenchymal stromal cell-derived extracellular vesicles. Bioact. Mater..

[171] Xu Y.Q., Zhang Z.H., Zheng Y.F., Feng S.Q. (2016). MicroRNA-221 Regulates Hypertrophy of Ligamentum Flavum in Lumbar Spinal Stenosis by Targeting TIMP-2. Spine.

[172] Wawrose R.A., Oyekan A.A., Tang Y.M., Chen S.R., Chen J., Couch B.K., Wang D., Alexander P.G., Sowa G.A., Vo N.V. (2024). MicroRNA-29a: A novel target for non-operative management of symptomatic lumbar spinal stenosis. Eur. Spine J..

[173] Ao X., Wang L., Shao Y., Chen X., Zhang J., Chu J., Jiang T., Zhang Z., Huang M. (2019). Development and Characterization of a Novel Bipedal Standing Mouse Model of Intervertebral Disc and Facet Joint Degeneration. Clin. Orthop. Relat. Res..

[174] Zheng Z.Y., Li P., Ao X., Qian L., Peng Y.X., Chu J., Jiang T., Lian Z.N., Zhang Z.M., Wang L. (2021). Characterization of a Novel Model of Lumbar Ligamentum Flavum Hypertrophy in Bipedal Standing Mice. Orthop. Surg..

[175] Wang B., Gao C., Zhang P., Sun W., Zhang J., Gao J. (2021). The increased motion of lumbar induces ligamentum flavum hypertrophy in a rat model. BMC Musculoskelet. Disord..

[176] Hsu Y.C., Chuang H.C., Tsai K.L., Tu T.Y., Shyong Y.J., Kuo C.H., Liu Y.F., Shih S.S., Lin C.L. (2022). Administration of N-Acetylcysteine to Regress the Fibrogenic and Proinflammatory Effects of Oxidative Stress in Hypertrophic Ligamentum Flavum Cells. Oxid. Med. Cell Longev..

[177] Deepmala, Slattery J., Kumar N., Delhey L., Berk M., Dean O., Spielholz C., Frye R. (2015). Clinical trials of N-acetylcysteine in psychiatry and neurology: A systematic review. Neurosci. Biobehav. Rev..

[178] Lauterburg B.H., Corcoran G.B., Mitchell J.R. (1983). Mechanism of action of N-acetylcysteine in the protection against the hepatotoxicity of acetaminophen in rats in vivo. J. Clin. Investig..

[179] Forman H.J., Zhang H. (2021). Targeting oxidative stress in disease: Promise and limitations of antioxidant therapy. Nat. Rev. Drug Discov..

[180] Darenskaya M.A., Kolesnikova L.I., Kolesnikov S.I. (2021). Oxidative Stress: Pathogenetic Role in Diabetes Mellitus and Its Complications and Therapeutic Approaches to Correction. Bull. Exp. Biol. Med..

[181] Yurube T., Buchser W.J., Zhang Z., Silwal P., Lotze M.T., Kang J.D., Sowa G.A., Vo N.V. (2024). Rapamycin mitigates inflammation-mediated disc matrix homeostatic imbalance by inhibiting mTORC1 and inducing autophagy through Akt activation. JOR Spine.

[182] Wu L., Xu L., Chen Y., Xu G., Guo Q., Meng D., Fan J., Song G., Xu P. (2021). Rolipram plays an anti-fibrotic effect in ligamentum flavum fibroblasts by inhibiting the activation of ERK1/2. BMC Musculoskelet. Disord..

